# Immunotoxin WPD101a as a Potential Drug Candidate for Targeted Therapy in Muscle Invasive Bladder Cancer Expressing IL-13Rα2—In Vitro Study

**DOI:** 10.3390/ijms27125566

**Published:** 2026-06-19

**Authors:** Aleksandra Klimczak, Agnieszka Krawczenko, Sandra Stamnitz, Aleksandra Bielawska-Pohl, Paulina Piotrowska, Hanna Grzelenska, Aleksandra Wypychowska, Alicja Kisielewicz, Marcin Mielecki, Radoslaw Borowski, Mariusz Olejniczak, Beata Pajak-Tarnacka

**Affiliations:** 1 Laboratory of Biology of Stem and Neoplastic Cells, Hirszfeld Institute of Immunology and Experimental Therapy, Polish Academy of Sciences, 53-114 Wroclaw, Poland; agnieszka.krawczenko@hirszfeld.pl (A.K.); sandra.stamnitz@hirszfeld.pl (S.S.); aleksandra.bielawska-pohl@hirszfeld.pl (A.B.-P.); paulina.piotrowska@hirszfeld.pl (P.P.); hanna.grzelenska@icloud.com (H.G.);; 2 WPD Pharmaceuticals, 02-089 Warsaw, Polandbeata.pajak@wpdpharmaceuticals.com (B.P.-T.)

**Keywords:** bladder cancer, muscle-invasive bladder cancer, MIBC, interleukin 13 receptor subunit alpha 2, IL-13Rα2, immunotoxin, targeted therapy

## Abstract

The failure of therapy in muscle invasive bladder cancer (MIBC) is primarily attributed to tumor heterogeneity and therapy resistance. We propose a novel approach targeting interleukin-13 receptor subunit alpha 2 (IL-13Rα2), which is expressed on bladder cancer (BC) cells but absent in normal urothelial cells. We investigated the therapeutic effects of WPD101a immunotoxin (IL-13-DT390) on IL-13Rα2-expressing BC cells in relation to BC cell phenotype and functional characteristics in vitro using both 2-dimensional (2D) and 3-dimensional (3D) models. Cell phenotype and IL-13Rα2 expression were assessed using flow cytometry, immunofluorescence, and Western blot analysis. The biological effects of WPD101a were evaluated by measuring cell viability and proliferation using the MTT, sulforhodamine B (SRB), CellTiter-Glo and Live/Dead assays. Apoptosis was assessed using Annexin V/propidium iodide (PI) staining, and quantitative reverse transcription polymerase chain reaction (qRT-PCR) analysis of *CASP* genes expression. We found that the reference BC cell lines TCC-SUP, JMSU-1 and UM-UC-3 express IL-13Rα2 at various level in contrast to RT-4, HCV-29 and 5637 cells. Cells expressing IL-13Rα2 were sensitive to WPD101a at lower concentrations in the 2D model (0.1 ng/mL) compared to the 3D model (1.0 ng/mL). IL-13Rα2-negative cells remain resistant to the immunotoxin. WPD101a induces apoptosis in BC cells expressing IL-13Rα2 as confirmed by the presence of apoptotic cells, increase the proportion of cells in the subG1 phase, and by the effector *CASP3*, *CASP7*, and initiator *CASP8*, *CASP9* genes expression. This study confirmed receptor-dependent cytotoxic effects of WPD101a and the ability and specificity to inhibit growth and apoptosis induction in MIBC cells expressing IL-13Rα2.

## 1. Introduction

Bladder cancer (BC) is the tenth most common malignant tumor of the urinary system and encompasses a broad clinical spectrum, ranging from localized non-muscle invasive lesion to advanced or metastatic disease [1]. The majority of urothelial cancers are diagnosed as non-invasive bladder cancer (NMIBC); however, approximately 25% of patients develop muscle-invasive bladder cancer (MIBC) and the estimated 5-year overall survival (OS) is approximately 50% [2].

MIBC is usually treated with transurethral resection followed by chemotherapy, depending on the histological grade tumor-node-metastasis (TNM) stage. In patients with high-risk NMIBC, the recommended treatment after tumor resection is adjuvant immunotherapy using intravesical Bacillus Calmette-Guérin (BCG) instillations. Although this therapeutic procedure has been used for more than 30 years, only approximately 50% of patients derive significant benefit from the treatment [3].

The standard of care for non-metastatic MIBC is radical cystectomy combined with simultaneous pelvic lymph node dissection, which improves the 5-year OS to up to 60% (8, 9). Due to the high risk of distant metastasis and mortality, neoadjuvant cisplatin-based chemotherapy is currently recommended to achieve better local and systemic disease control, according to both the National Comprehensive Cancer Network (NCCN) [4] and the European Association of Urology (EAU) guidelines [2].

Immunotherapy has been approved by the U.S. Food and Drug Administration (FDA) as a second-line treatment for metastatic BC and as maintenance therapy following cisplatin-based chemotherapy. Combination chemotherapy consisting of gemcitabine and immune checkpoint inhibitors of programmed death 1 (PD-1 or PD-L1), such as pembrolizumab, nivolumab, durvalumab, and atezolizumab have been evaluated in non-randomized clinical trials. Although these studies demonstrated downstaging of the disease, the lack of randomization limits the ability to predict long-term disease-free or overall survival outcomes [5].

Unsatisfactory treatment outcomes have prompted the search for novel therapeutic approaches based on the biological properties of the tumor. Accumulating evidence suggests that a subpopulation of tumor cells, known as cancer stem cells (CSCs), characterized by stem-like properties such as self-renewal and multipotency, is responsible for tumor initiation, proliferation, invasive growth, tumor heterogeneity and metastasis formation. CSCs are also considered one of the major causes of therapy failure in MIBC [6]. Due to the high heterogeneity of the CSCs, no specific markers for BC CSCs have been definitively established. However, the expression of CD133 has been significantly correlated with poorer recurrence-free survival in BC patients. Moreover, CD44, a prominent stem cell marker expressed on cells located in the basal layer of both normal and tumor urothelium, as well as ALDH1 expression, has been correlated with distant tumor metastasis [7,8]. The presence of BC cells with a specific stem-like phenotype may therefor contribute to tumor recurrence and metastatic progression.

Due to the high risk of distant metastasis and mortality, MIBC is treated aggressively; however, unsatisfactory therapeutic outcomes have prompted the search for novel treatment approaches based on the biological properties of the tumor. One potential therapeutic target that has been recently gained considerable attention in cancer therapy is IL-13Rα2, a membrane receptor for the IL-13.

IL-13Rα2 was initially considered a decoy receptor that sequesters IL-13 and inhibits its signaling. However, recent studies have demonstrated that IL-13-mediated IL-13Rα2 signaling occurs through STAT6-independent pathways, involving activation of activator protein 1 (AP-1) and extracellular signal-related kinase (ERK), thereby promoting tumor invasion, metastasis, and production of transforming growth factor beta (TGF-β) [9]. IL-13Rα2 is overexpressed in various cancer types and has been shown to correlate with advanced disease and poor prognosis [10,11]. The most advanced studies have been performed in glioblastoma (GBM) models, as IL-13Rα2 is overexpressed in approximately 76% of GBM cases but is not detected in normal brain tissue, making it a highly selective target for immunotherapy [10,12]. According to the Human Protein Atlas database, IL-13Rα2 overexpression has also been detected in BC specimens [13] (accessed 28 April 2026). Moreover, recent multi-omics analyses revealed significant overexpression of IL13Rα2 in urothelial carcinoma, where it appears to promote pro-inflammatory signaling, myeloid cell recruitment, and resistance to immune checkpoint blockade [14]. Thus, the evaluation of IL13-Rα2-directed treatment should be verified in MIBC indication.

One of the approaches in the development of targeted therapies directed against IL-13Rα2 involves the use of immunotoxins, which are fusion biotherapeutics composed of two main components: a receptor-binding moiety and a cytotoxic payload. The targeting component is typically an antibody or ligand specific to a receptor expressed on the cell surface, such as IL-13Rα2. The effector component consists of a toxic agent, including drugs, radioisotopes, enzymes, or protein toxins [14,15].

In the case of WPD101a, the targeting moiety is recombinant IL-13, which exhibits high affinity for the IL-13Rα2 receptor. To increase affinity toward the IL-13RA2 receptor while reducing nonspecific interactions with the physiological IL13RA1 receptor complex, two point mutations (E13K and R66D) were introduced into the IL-13 sequence, as previously described by Waldemar Debinski and colleagues [16]. The cytotoxic component is a truncated form of diphtheria toxin (DT390), which, upon internalization into the cell, inhibits protein synthesis and induces apoptosis [17]. The cytotoxic activity of WPD101a (IL-13–DT390) has previously been validated in a canine glioma model [18]; however, to date, no studies have investigated this therapeutic approach in BC models.

At present, targeted therapy for MIBC is very limited, and the identification of new targets for therapeutic intervention will be promising tool in MIBC treatment modalities. Targeted therapies interfering with cellular processes crucial for cancer growth have been introduced as a novel treatment modality over the past two decades [19], but to date, no targeted agent has been approved for BC treatment. As a new target for therapeutic intervention, we propose IL-13Rα2, which is expressed on BC cells but not on normal urothelial cells.

## 2. Results

The biological activity of the immunotoxin WPD101a was assessed using 2D and 3D culture models of BC reference cell lines: the benign HCV-29 bladder epithelial cell line, low-grade RT-4 and high-grade 5637, TCC-SUP, JMSU-1 and UM-UC-3, and results are presented separately for each model. In addition to assessing activity, this approach enables a functional comparison of the two models, highlighting their respective advantages and limitations. Presenting the data in parallel provides a broader perspective on the biological effects of the investigated molecule. Accordingly, the results obtained in the 2D model are described first, followed by those from the 3D system.

### 2.1. The Analysis of BC Cell Lines for the Presence of Cells with Cancer Stem Cell (CSC) Phenotype and IL-13Ra2 Expression in 2D Model

#### 2.1.1. Phenotypic Characterization of BC Cell Lines by Flow Cytometry

**Figure 1:**
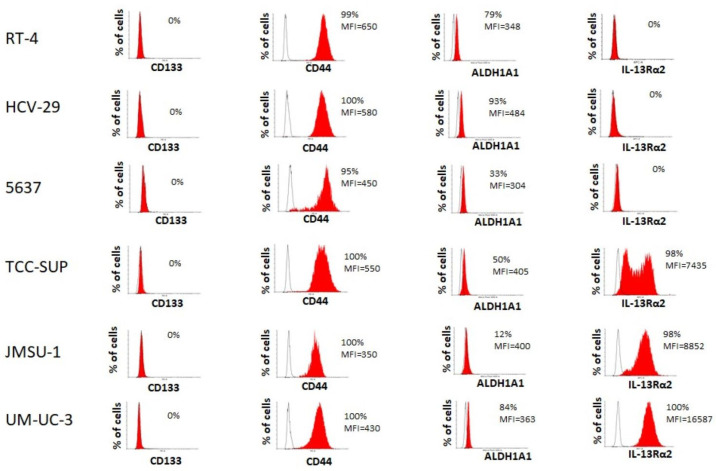
Representative flow cytometry histograms showing the phenotype of non-malignant (RT-4, HCV-29) and malignant (5637, TCC-SUP, JMSU-1 and UM-UC-3) BC cell lines for the expression of CD44, CD133, ALDH1A1 and IL-13Rα2, and median fluorescence intensity (MFI) assessment. Red histograms illustrate the percentage of analyzed markers, while empty histograms isotypic controls.

To assess the biological features of BC cell lines, cells were cultured under 2D conditions and stained with antibodies against the surface markers CD133 and CD44, as well as the intracellular marker ALDH1A1. The expression level of IL-13Rα2, the membrane target of the immunotoxin WPD101a, was also evaluated. The results are presented in Figure 1 and Table 1.

In the analyzed reference BC cell lines, no expression of CD133 was detected, whereas CD44 expression was observed in all lines. In addition, a high percentage of ALDH1-positive cells was found in RT-4, HCV-29, and UM-UC-3 cells (79%, 93% and 84% respectively), a moderate percentage in the TCC-SUP line (50%), and a low percentage in the 5637 and JMSU-1 lines (33% and 12%).

In parallel, RT-4, HCV-29, and 5637 cells were confirmed to lack IL-13Rα2 on the cell surface, whereas TCC-SUP, JMSU-1, and UM-UC-3 cell lines exhibited IL-13Rα2 expression at varying levels. The lowest expression was observed in TCC-SUP cells (MFI = 7435), intermediate expression in JMSU-1 cells (MFI = 8852), and the highest expression in UM-UC-3 cells (MFI = 16,587). Furthermore, two distinct subpopulations were identified within the TCC-SUP cell line, characterized by low (IL-13Rα2low) and high (IL-13Rα2high) levels of IL-13Rα2 expression (Table 1).

#### 2.1.2. Validation of Phenotypic Markers of BC Cell Lines by Western Blot and Immunofluorescence Analysis

**Figure 2:**
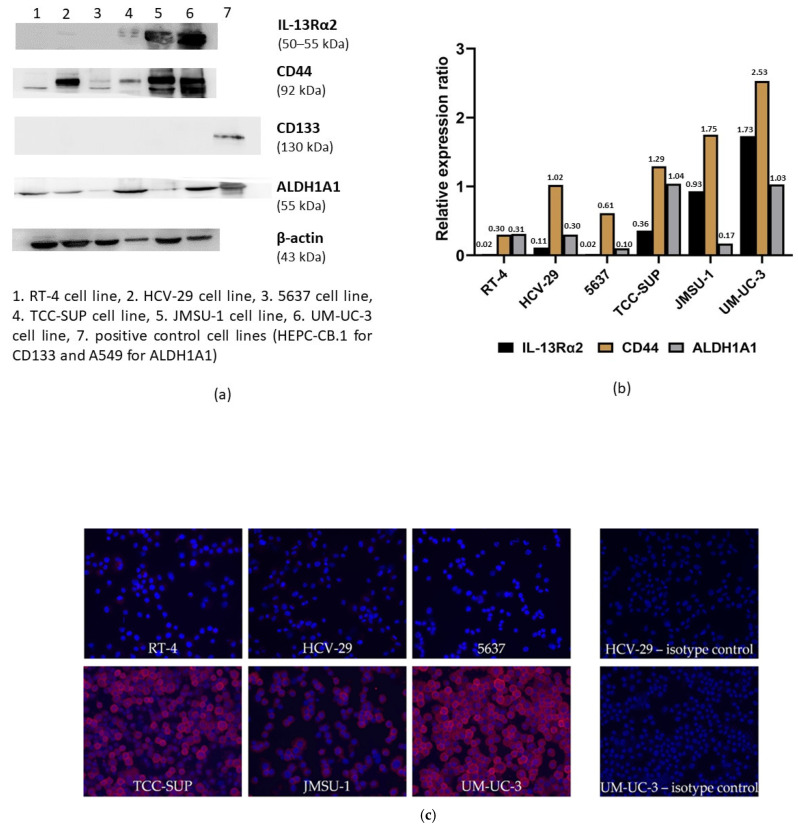
Analysis of the expression levels of IL-13Rα2, CD44, CD133, ALDH1A1, and β-actin in lysates of commercial BC cell lines cultured in a 2D model, determined by WB: (**a**) chemiluminescent image, (**b**) densitometric analysis of the obtained results performed using ImageJ V1.54 software, (**c**) immunofluorescence staining for IL-13Rα2 expression. Cell nuclei were stained with DAPI (blue) and IL-13Rα2 expression was visualized with AF647 (red).

In the next step, Western blot (WB) analysis was performed to confirm the results determining the expression levels of the selected molecules in commercial BC cell lines. The results of the analyses are presented in Figure 2a,b (original WB gel is presented in Figure S1).

The WB results confirmed the absence of IL-13Rα2 expression in RT-4 and 5637 cell lines and its presence in TCC-SUP, JMSU-1, and UM-UC-3 cell lines. However, a very low level of IL-13Rα2 expression was observed in HCV-29 cells, which was not detectable by flow cytometry. CD44 was expressed in all examined cell lines, while the absence of CD133 expression was confirmed in all tested BC cell lines. ALDH1A1 was highly expressed in UM-UC-3, TCC-SUP, and RT-4 cell lines, whereas low expression levels were detected in HCV-29, 5637, and JMSU-1 cell lines in 2D cellular model.

Additionally, immunofluorescence staining for IL-13Rα2 confirmed its presence in TCC-SUP, JMSU-1 and UM-UC-3 cells and its absence in the RT-4, HCV-29 and 5637 cell lines. Two cell populations with high and low IL-13Rα2 expression were observed in TCC-SUP cells, consistent with the flow cytometry analysis (Figure 2c).

The results indicate that BC cell line do not express CD133, as confirmed by both flow cytometry and WB analysis. All tested cell lines expressed CD44 and ALDH1A1, markers associated with tumor aggressiveness and distant metastasis. High levels of IL-13Rα2 expression were detected in the JMSU-1, and UM-UC-3 cell lines; however, in the TCC-SUP cell line, two populations of cells with high and low IL-13Rα2 expression were identified. Although IL-13Rα2 expression was not detected in the HCV-29 cell line by flow cytometry, a very low expression level was identified by WB.

### 2.2. Functional Assessment of BC Cells Exposed to the Immunotoxin WPD101a

Cytotoxicity and proliferation assays in 2D culture model were performed on all tested BC cell lines: RT-4, HCV-29, 5637, TCC-SUP, JMSU-1 and UM-UC-3. Cells were treated with WPD101a immunotoxin at concentrations ranging from 0.01–10.0 ng/mL for 24, 48 and 72 h.

#### 2.2.1. Cytotoxicity of the Immunotoxin WPD101a Against BC Cells

**Figure 3:**
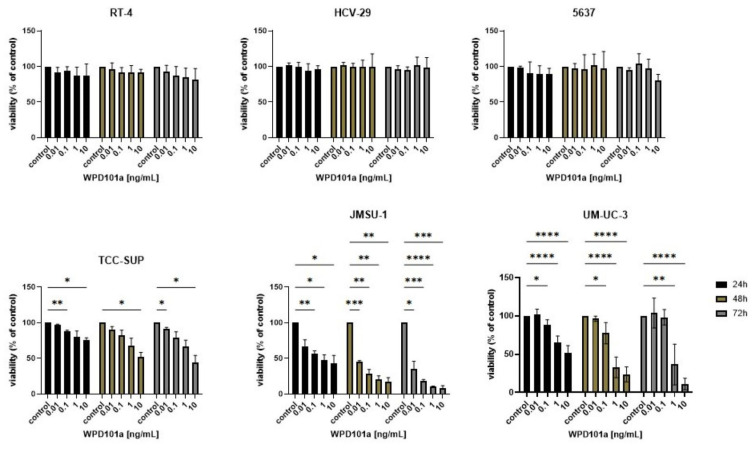
Cytotoxicity of immunotoxin WPD101a. BC cells were cultured in the presence of the immunotoxin WPD101a at concentrations range of 0.01–10.0 ng/mL. After 24, 48 and 72 h MTT assay was performed. Untreated cells were used as a control. Results are presented as the mean of at least three independent experiments ± SD. Statistical significances are indicated as follow: *p* < 0.05 (*), *p* < 0.01 (**), *p* < 0.001 (***), and *p* < 0.0001 (****).

For cytotoxicity assessment, the MTT assay was performed, and the results are shown in Figure 3. HCV-29, RT-4, and 5637 cells did not show sensitivity to the immunotoxin. In contrast, exposure of TCC-SUP cells to 10.0 ng/mL of WPD101a resulted in a reduction in the number of viable cells by more than 50% after 72 h. JMSU-1 cells proved to be the most sensitive to the immunotoxin, with a decrease in the percentage of proliferating cells observed after only 24 h across the entire range of tested WPD101a doses (0.01–10.0 ng/mL), from 67.1% at 0.01 ng/mL (*p* = 0.0083) to 43.5% at 10.0 ng/mL (*p* = 0.0251). UM-UC-3 cells showed similar sensitivity at concentrations of 1.0 and 10.0 ng/mL, the percentage of proliferating cells was 51.8% after 24 h, (*p* < 0.0001) and 11.2% after 72 h (*p* < 0.0001).

#### 2.2.2. The Effect of WPD101a Immunotoxin Treatment on BC Cells Proliferation

**Figure 4:**
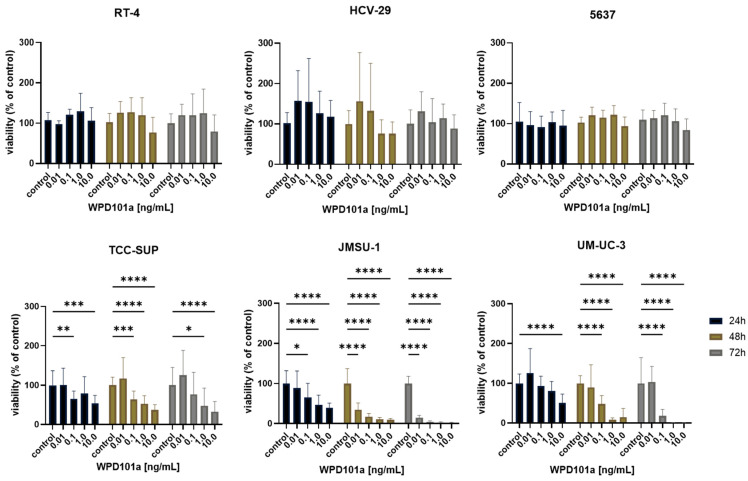
Proliferation of BC cells after WPD101a treatment. Cells are cultured in the presence of the immunotoxin WPD101a at concentrations range of 0.01–10.0 ng/mL. After 24, 48 and 72 h SRB assay was performed. Untreated cells were used as a control. Results are presented as the mean of at least three independent experiments ± SD. Statistical significances are indicated as follow: *p* < 0.05 (*), *p* < 0.01 (**), *p* < 0.001 (****), and *p* < 0.0001 (****).

For the assessment of cells proliferation, the SRB assay was performed, and the results are shown in Figure 4. Similarly to the cytotoxicity assay, the most sensitive BC cell line was JMSU-1. After only 24 h of WPD101a treatment, a significant decrease in the percentage of proliferating cells was observed (from 65.4% for 0.1 ng/mL WPD101a, *p* = 0.0282 to 39.6% for 10.0 ng/mL, *p* < 0.0001). After 48 and 72 h, the percentage of proliferating cells decreased to very low levels (4.1% at 0.1 ng/mL WPD101a, *p* < 0.0001, to 1.8% at 10.0 ng/mL WPD101a, *p* < 0.0001, after 72 h).

UM-UC-3 cells also showed a significant decrease in proliferation, particularly at concentrations ranging from 0.1 ng/mL to 10.0 ng/mL. This effect was clearly visible after 48 and 72 h of treatment; after 48 h proliferation decreased below 50% of control levels (47.8% at 0.1 ng/mL WPD101a, *p* < 0.0001, and 14.8% at 10.0 ng/mL WPD101a, *p* < 0.0001). After 72 h of WPD101a treatment, almost complete inhibition of UM-UC-3 cell proliferation was observed at concentrations of 1.0 ng/mL (0.6%, *p* < 0.0001) and 10.0 mg/mL (0.2%, *p* < 0.0001).

A decrease in proliferation was also observed in TCC-SUP cells at WPD101a concentrations of 0.1, 1.0 and 10.0 ng/mL; however, the effect was less pronounced compared with JMSU-1 and UM-UC-3 cells (from 47.5% for 1.0 ng/mL WPD101a, *p* = 0.0103, to 32.7% for 10.0 ng/mL WPD101a, *p* < 0.0001, after 72 h).

### 2.3. Determination of Inhibitory Concentration (IC_50_) in 2D Culture

**Figure 5:**
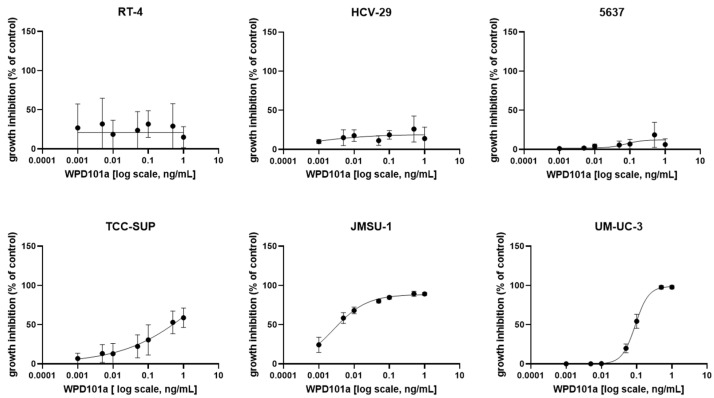
Determination of IC_50_. BC cell lines were cultured for 72 h with appropriate concentrations (0.001 to 10.0 ng/mL) of the WPD101a immunotoxin followed by the MTT assay. Untreated cells served as a control (100%) and results were calculated as a percentage of inhibition. Results are presented as the mean of at least three independent experiments ± SD.

For IC_50_ determination, cells were cultured with appropriate concentrations of the WPD101a immunotoxin ranging from 0.001 to 1.0 ng/mL, followed by the MTT assay. The results are presented in Table 2 and Figure 5.

For UM-UC-3 cells, the IC_50_ value of the WPD101a immunotoxin was determined to be 0.09154 ng/mL, for JMSU-1 cells 0.002655 ng/mL, and for TCC-SUP cells 3.311 ng/mL. The remaining BC cell lines were not sensitive to WPD101a.

### 2.4. Analysis of the Sensitivity of BC Cell Lines to the Immunotoxin WPD101a Performed by Flow Cytometry Using Annexin V and Propidium Iodide (PI) Staining for Cells Grown in 2D Cultures

**Figure 6:**
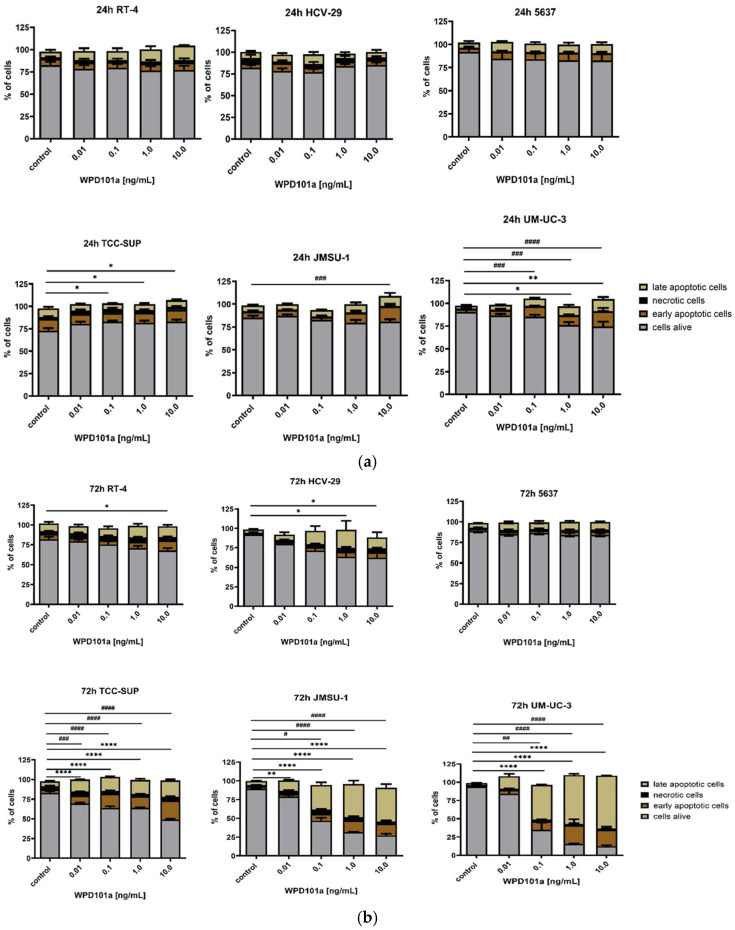
Percentage of early and late apoptotic, necrotic, and viable cells in 2D cultures of BC cell lines treated with the immunotoxin at concentrations of 0.01, 0.1, 1.0, and 10.0 ng/mL, as well as untreated control cells (control), after (**a**) 24 and (**b**) 72 h of exposure to WPD101a. Results are presented as the mean of at least three independent experiments ± SD. Statistical significance for viable cells (*) and apoptotic cells (#) was determined using one-way analysis of variance (ANOVA) followed by Dunnett’s post hoc test (*p* < 0.05 (*, #), *p* < 0.01 (**, ##), *p* < 0.001 (###), *p* < 0.0001 (****, ####).

Subsequently, an apoptosis assay was performed in BC cells cultured in the presence of the immunotoxin at WPD101a, and results are presented in Figure 6.

After 24 h of incubation, changes in sensitivity to WPD101a were observed only in the JMSU-1 and UM-UC-3 cell lines at concentrations of 1.0 and 10.0 ng/mL. In both cases, a slight, non-significant decrease in the percentage of viable cells compared with the control was observed. In JMSU1 cells, a statistically significant increase in the proportion of early apoptotic cells was detected at 1.0 and 10.0 ng/mL, rising from 6.5% in the control to 10.6% and 16.7%, respectively. In contrast, UM-UC-3 cells exhibited a significant increase in late apoptotic cells at 0.1, 1.0, and 10.0 ng/mL (8.1%, 9.6%, and 13.4%, respectively), compared with 3.2% in the control (Figure 6a).

More pronounced and statistically significant effects were observed after 72 h of incubation (Figure 6b). In the HCV-29 cell line, a significant response was detected only at 10.0 ng/mL, manifested by an increase in early apoptotic cells from 1.1% (control) to 6.6%, and an increase in necrotic cells from 1% to 5.6%. In contrast, prolonged incubation revealed marked sensitivity to the immunotoxin in the TCC-SUP, JMSU-1, and UM-UC-3 cell lines across all tested concentrations. In TCC-SUP cells, the percentage of viable cells decreased from 83% in the control to 70%, 64%, 64%, and 49%, respectively, with increasing immunotoxin concentrations. This decrease was accompanied by a significant increase in apoptotic cells. The proportion of early apoptotic cells increased from 4% in the control to 10%, 18%, 14%, and 24%, while late apoptotic cells increased from 6% to 15%, 18%, 18%, and 21%, respectively.

Similarly, JMSU-1 cells exhibited a pronounced reduction in cell viability, decreasing from 89% in the control to 47%, 32%, and 27% at 0.1, 1.0, and 10.0 ng/mL, respectively. This effect was associated with a significant increase in apoptosis. The proportion of early apoptotic cells increased from 3% in the control to 9%, 15%, and 16% at concentrations ≥ 0.1 ng/mL. Concurrently, the proportion of late apoptotic cells increased progressively across all concentrations, from 5% in the control to 14%, 33%, 44%, and 45%.

Treatment of UM-UC-3 cells resulted in a significant, dose-dependent reduction in viability, decreasing from 94% in the control to 16% and 12% at 1.00 and 10.00 ng/mL, respectively. This reduction was strongly correlated with elevated levels of apoptosis. The proportion of early apoptotic cells increased from 1% in the control to 12%, 25%, and 22% (at 0.1, 1.0, and 10.0 ng/mL, respectively), while the proportion of late apoptotic cells increased progressively, reaching up to 72% at the highest concentration.

The results showed that treatment with the WPD101a immunotoxin induces apoptosis in BC cells expressing IL-13Rα2 (UM-UC-3, JMSU-1, TCC-SUP), but not in IL-13Rα2-negative cells (5637, RT-4), as confirmed by the presence of cells in the early and late stages of apoptosis. The percentage of apoptotic cells elevated significantly with increasing doses of WPD101a and with prolonged incubation from 24 to 72 h.

#### 2.4.1. Analysis of the Sensitivity of BC Cell Lines to the Immunotoxin WPD101a Using Flow Cytometry with a Cell Cycle Phase Distribution Assay in 2D Model

**Figure 7:**
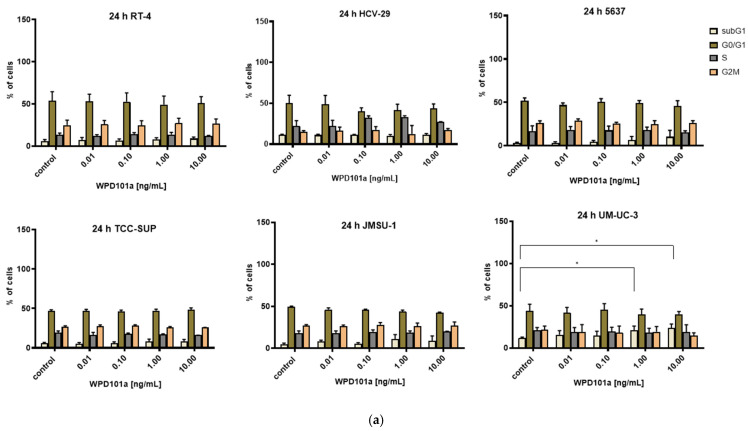

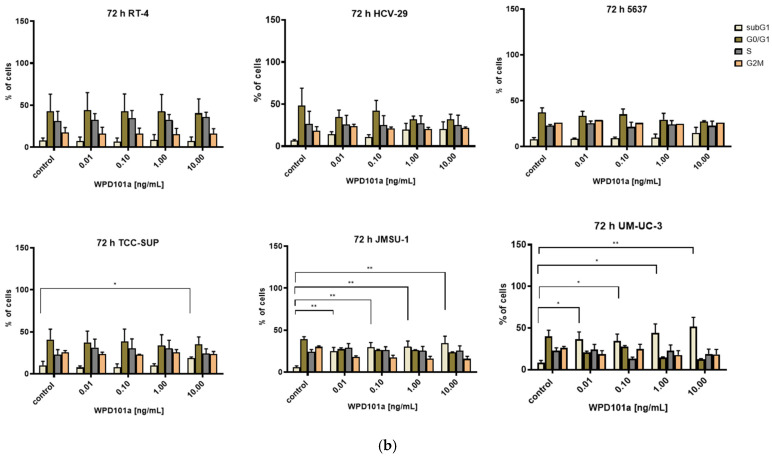
Percentage of cells in individual phases of the cell cycle in 2D cultures of BC cell lines treated with the WPD101a immunotoxin at concentrations of 0.01, 0.1, 1.0, and 10.0 ng/mL, as well as control cells (control), after (**a**) 24 h and (**b**) 72 h [% ± SD]. Results are presented as the mean of at least three independent experiments ± SD. Statistical significances are indicated as follow: *p* < 0.05 (*), *p* < 0.01 (**) was determined using two-way analysis of variance (ANOVA) with Dunnett’s multiple comparison test.

The percentage of cells in individual phases of the cell cycle in BC cell lines treated with the immunotoxin after 24 and 72 h of exposure is shown in Figure 7.

Cell cycle analysis showed that the immunotoxin did not arrest BC cells in any specific phase of the cell cycle. However, the results confirmed that WPD101a induced apoptosis, as evidenced by a statistically significant increase in the proportion of cells in the subG1 phase. After 24 h, this effect was observed only in UM-UC-3 cells at doses of 1.0 and 10.0 ng/mL, with the subG1 population increasing from 7% in the control to 20% and 23%, respectively.

After 72 h of incubation with WPD101a, both JMSU1 and UM-UC-3 cell lines exhibited a statistically significant increase in the subG1 population at all tested concentrations compared to controls. In JMSU-1 cells, the subG1 fraction increased from 7% in the control to 24%, 29%, 31%, and 34% at WPD101a concentrations of 0.01, 0.1, 1.0, and 10.0 ng/mL, respectively. In UM-UC-3 cells, the subG1 fraction increased from 5% in the control to 36%, 34%, 44%, and 52% at the same concentrations. Furthermore, prolonged incubation (72 h) induced a statistically significant increase in subG1 cells in the TCC-SUP line, but only at the highest tested dose of 10.0 ng/mL, with an increase from 10% in the control to 16% in treated samples.

A significant increase in the proportion of BC cells, expressing IL-13Ra2 in the subG1 phase was confirmed after 72 h of incubation with WPD101a. These findings are consistent with the results obtained from apoptosis assays using Annexin V/PI staining and flow cytometry and further support the sensitivity of BC cell lines expressing IL-13Ra2 to the WPD101a immunotoxin.

#### 2.4.2. Expression of Genes Associated with Cell Apoptosis After WPD101a Immunotoxin Treatment

**Figure 8:**
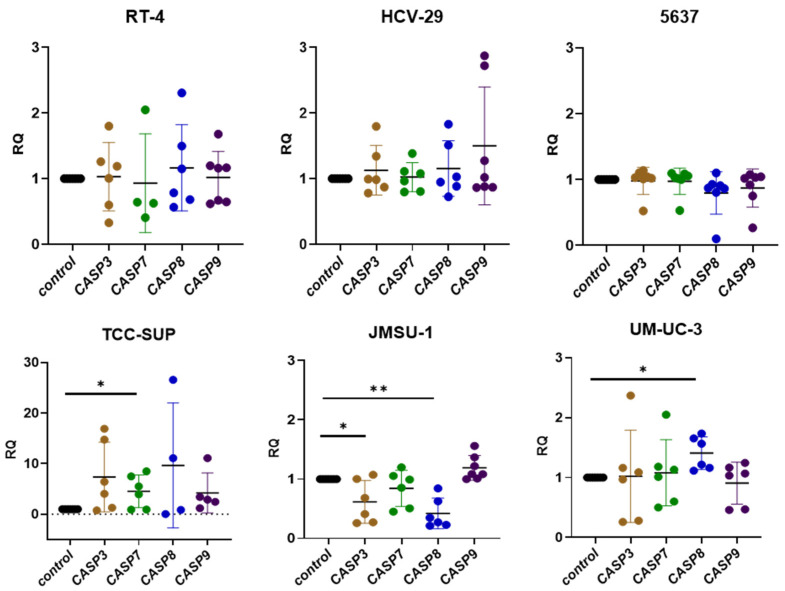
Expression of genes associated with cell apoptosis. BC cell lines were cultured for 72 h in the presence of the immunotoxin WPD101a at a concentration of 0.1 ng/mL. *CASP3*, *7*, *8* and *9* gene expression was determined by qRT-PCR method with GADPH used as the reference gene. The results were analyzed by the ΔΔCt method and expressed as RQ, with untreated cells used as the control. Data represents mean ± SD, N ≥ 4; *p* < 0.05 (*), *p* < 0.01 (**).

BC cell lines were cultured for 72 h in the presence of the WPD101a immunotoxin at a concentration of 0.1 ng/mL. Caspase 3, 7, 8 and 9 gene expression levels were determined using quantitative real-time PCR method (qRT-PCR). The results are presented in Figure 8 as fold change relative to the untreated control cells.

The results showed a statistically significant increase in *CASP8* expression (*p* = 0.0148) and no changes in *CASP 3*, *7* and *9* expression in UM-UC-3 cells. In JMSU-1 cells, a significant decrease in *CASP3* (*p* = 0.0483) and *CASP8* (*p* = 0.0027) gene expression was observed, with no changes in *CASP7* and *CASP9* gene expression after treatment with WPD101a. In RT-4, HCV-29, and 5637 cells, no significant changes in the expression of the examined caspase genes were detected after treatment with the immunotoxin WPD101a. In TCC-SUP cells, a statistically significant increase in *CASP7* expression only (*p* = 0.0436) was observed.

### 2.5. WPD101a Activity in a 2D BC Model Assessed by IL-13Rα2 Expression Using Immunofluorescence Staining

**Figure 9:**
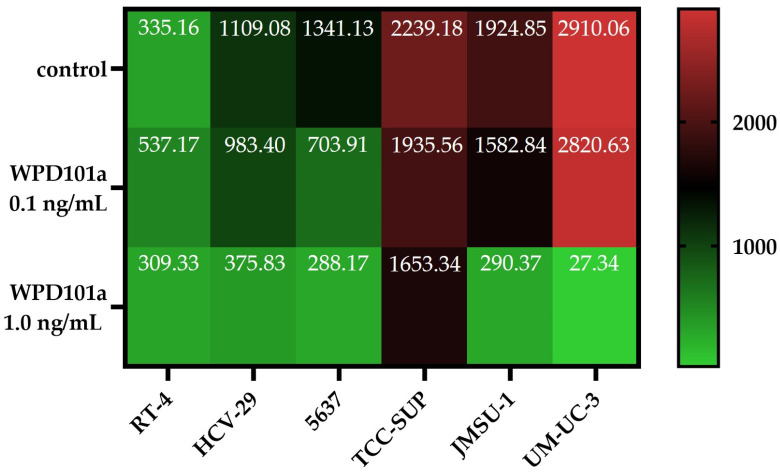
Effect of two concentrations of WPD101a (0.1 ng/mL and 1.0 ng/mL) on BC cell lines. Heatmap showing the MFI values measured for IL-13Rα2 receptor expression under all tested conditions. Immunofluorescence staining was performed in duplicate. The microscopic images of the immunofluorescence staining are presented in Figure S2.

The RT-4 cell line showed no detectable expression of IL-13Rα2 in either the control or treated groups, as demonstrated by the immunofluorescence imaging (Figure S2) and confirmed by the mean fluorescence intensity (MFI) of approximately ~310 shown in the composed heatmap (Figure 9). In the HCV-29 and 5637 cell lines, a slight increase in IL-13Rα2 receptor expression was observed in the control groups, with MFI of 1109.07 and 1341.13, respectively (Figure 9). Interestingly, in 5637 cells, the fluorescence signal originated mainly from chromosomes in mitotic cells, with no visible signal on the cell membrane or in the cytoplasm. For these three cell lines, no effect of WPD101a was observed, regardless of the concentration, and no changes in cell number were detected.

The TCC-SUP line exhibited IL-13Rα2 expression in the control group (Figure S2) that was almost twice as high as that observed in HCV-29 and 5637 cells, with an MFI of 2239.18 (Figure 9). Furthermore, TCC-SUP cells cultured in the presence of WPD101a showed a decrease in MFI to 1935.56 at 0.1 ng/mL WPD101a and 1653.34 at 1.0 ng/mL WPD101a. In the JMSU-1 cell line, a reduction in the MFI was also observed in cells cultured with 0.1 ng/mL WPD101a (MFI = 1582.84) compared with the control (MFI = 1924.85). Interestingly, at a higher concentration of WPD101a (1.0 ng/mL), a marked reduction in cell number was observed, resulting in an MFI of only 290.37 (Figure 9).

The highest expression of IL-13Rα2 was detected in UM-UC-3 cells, which, under standard culture conditions, exhibited an MFI of 2910.06 (Figure 9). However, treatment with WPD101a at 0.1 ng/mL resulted in a substantial reduction in cell number, accompanied by a slight decrease in MFI to 2820.63. The strongest effect in this cell line was observed at 1.0 ng/mL WPD101a, where the MFI decreased to 27.33, the lowest value recorded (Figure 9).

### 2.6. Functional Assessment of WPD101a in 3D BC Model

To verify the effect of WPD101a on cytotoxicity and cell proliferation in a 3D model, spheroids derived from six BC cell lines-RT-4, HCV-29, 5637, TCC-SUP, JMSU-1, and UM-UC-3, were treated with WPD101a at concentrations ranging from 0.1 to 10.0 ng/mL for 72 h.

#### 2.6.1. Cytotoxicity of WPD101a in 3D BC Model

WPD101a did not significantly affect the viability of RT-4, 5637, and HCV-29 spheroids, as their viability at all time points remained approximately 90–100% relative to the untreated control. In TCC-SUP spheroids, a decrease in viability was observed following treatment with 10.0 ng/mL WPD101a for 72 h, reaching 82.08% of viability.

**Figure 10:**
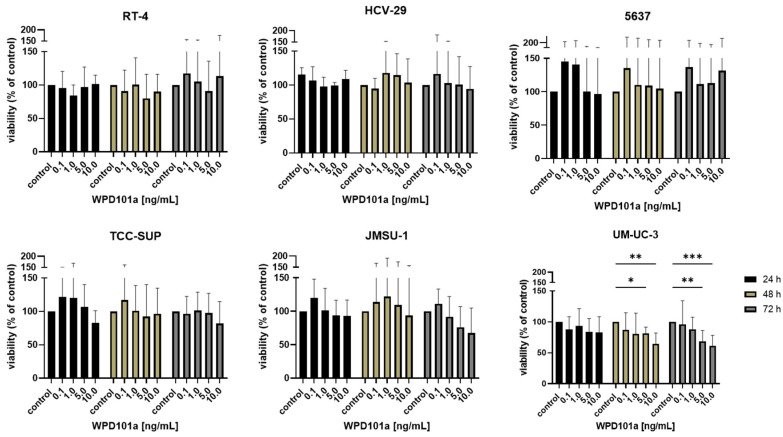
Effect of WPD101a on the viability of 3D BC spheroids. Spheroids derived from RT-4, HCV-29, 5637, TCC-SUP, JMSU-1, and UM-UC-3 cell lines were treated with increasing concentrations of WPD101a (0.1–10.0 ng/mL) for 24, 48, and 72 h. Cell viability was determined using the MTT assay and expressed as a percentage relative to untreated control spheroids (set as 100%). Results are presented as the mean of at least three independent experiments ± SD. Statistical significances are indicated as follow: *p* < 0.05 (*), *p* < 0.01 (**), *p* < 0.001 (***) was determined using two-way analysis of variance (ANOVA) with Dunnett’s multiple comparison test.

In contrast, higher cytotoxicity of WPD101a was observed in JMSU-1 and UM-UC-3 spheroids. After 72 h, treatment with 1.0 ng/mL WPD101a reduced JMSU-1 spheroid viability to 91.86%, while the highest concentration (10.0 ng/mL) decreased viability to 67.31%. Notably, UM-UC-3 spheroids were the most sensitive to WPD101a, with treatment at 10.0 ng/mL WPD101a reducing their number to 61.10% relative to the untreated control (Figure 10).

#### 2.6.2. Effect of WPD101a on Cell Viability Assessed by Live/Dead Staining

**Figure 11:**
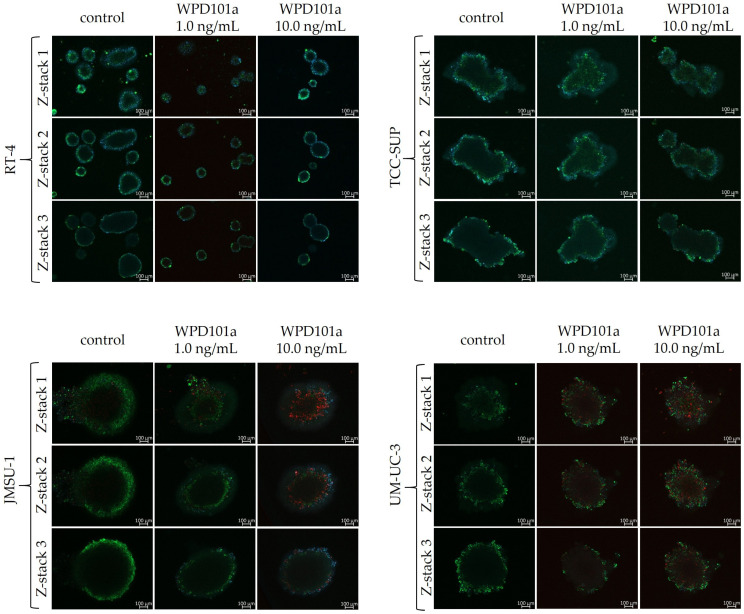
Live/Dead analysis of BC spheroids following WPD101a treatment. BC spheroids derived from RT-4, TCC SUP, JMSU 1, and UM-UC 3 cell lines were treated with WPD101a (1.0 ng/mL and 10.0 ng/mL) for 72 h or left untreated (control). Cell viability was assessed using the Live/Dead Cell Imaging Kit. Viable cells were stained with calcein-AM (green), whereas dead cells were labeled with PI (red). Nuclei were stained with DAPI (blue). Representative merged fluorescence images were generated from three different Z-stack and acquired using an Axio Observer microscope equipped with an Apotome module.

The Live/Dead fluorescence assay was performed to evaluate the effect of WPD101a on the viability of BC spheroids (Figure 11). In RT-4 spheroids, predominantly green fluorescence (derived from calcein-AM) was observed under all conditions, indicating a high proportion of viable cells. Treatment with WPD 101a at both 1.0 ng/mL and 10.0 ng/mL did not result in a noticeable increase in red fluorescence (derived from PI), suggesting lack of cytotoxic effect in this cell line. TCC-SUP spheroids also exhibited largely preserved viability following treatment. Although a slight increase in red fluorescence was detectable at 10.0 ng/mL, the overall structure and viability of the spheroids remained comparable to control, indicating only a moderate and limited induction of cell death. In contrast, JMSU-1 spheroids demonstrated a clear increase in red fluorescence following WPD101a exposure, particularly at the higher concentration. While control spheroids consisted predominantly of viable (green) cells, treatment with 10.0 ng/mL WPD101a resulted in a marked accumulation of dead (red) cells, indicating enhanced cytotoxicity and reduced proliferative capacity. The most pronounced effect was observed in UM-UC-3 spheroids. A substantial increase in red fluorescence was detected even at 1.0 ng/mL of WPD 101a and became more prominent at 10.0 ng/mL, suggesting a strong dose-dependent induction of cell death in this cell line.

#### 2.6.3. Effect of WPD101a on Proliferative Activity of BC Spheroids

**Figure 12:**
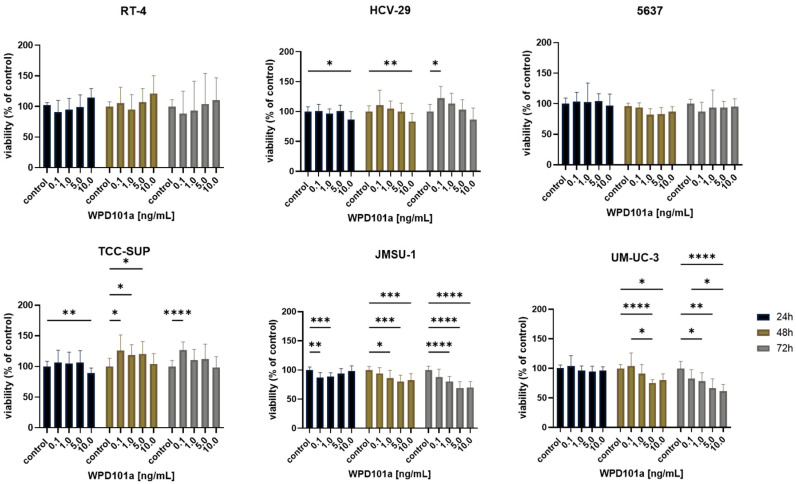
Influence of WPD101a on the proliferation of 3D BC spheroids. Spheroids generated from RT-4, HCV-29, 5637, TCC-SUP, JMSU-1, and UM-UC-3 cell lines were exposed to increasing concentrations of WPD101a (0.1–10 ng/mL) for 24, 48, and 72 h. Proliferative activity was assessed using the CellTiter-Glo 3D assay, and results were expressed as a percentage relative to untreated control spheroids, which were defined as 100%. Results are presented as the mean of at least three independent experiments ± SD. Statistical significances are indicated as follow: *p* < 0.05 (*), *p* < 0.01 (**), *p* < 0.001 (***), *p* < 0.0001 (****) was determined using two-way analysis of variance (ANOVA) with Dunnett’s multiple comparison test.

The effect of WPD101a on the proliferative activity of BC spheroids, assessed using the CellTiter-Glo 3D assay, is shown in Figure 12. In RT-4 and 5637 spheroids, WPD101a did not induce substantial changes in proliferation across the tested concentration range (0.1–10.0 ng/mL), with cell viability remaining close to control levels at all time points, indicating relative resistance to the compound. In contrast, HCV-29 spheroids exhibited moderate fluctuations in proliferative activity. While lower concentrations had minimal impact, higher doses and longer exposure times led to statistically significant changes, with cell viability decreasing to 86.01% following treatment with 10.0 ng/mL for 72 h.

TCC-SUP spheroids demonstrated a pronounced increase in proliferation at intermediate concentrations, particularly after 48 and 72 h, with several significant differences compared to the control. In JMSU-1 spheroids, short-term exposure to WPD101a resulted in limited effects, whereas prolonged treatment, especially at higher concentrations, significantly reduced proliferative activity, suggesting increased time-dependent sensitivity. Following 72 h of WPD101a treatment, the viability of JMSU 1 spheroids was reduced to 70%.

The most pronounced inhibitory effect was observed in UM-UC-3 spheroids. Treatment with WPD101a led to a clear dose-dependent decrease in proliferation, particularly after 72 h, with viability decreasing to 61.18% at the highest concentration tested (10.0 ng/mL).

Functional assays performed in the 3D spheroid model confirmed the sensitivity of IL-13Rα2-expressing BC cells to the WPD101a immunotoxin. In UM-UC-3 spheroids, a significant decrease in cell viability and a dose-dependent reduction in proliferation were observed. These findings confirm that UM-UC-3 cells, which exhibit the highest IL-13Rα2 expression, are the most sensitive to WPD101a treatment.

### 2.7. Determination of Inhibitory Concentration (IC_50_) in 3D Culture

**Figure 13:**
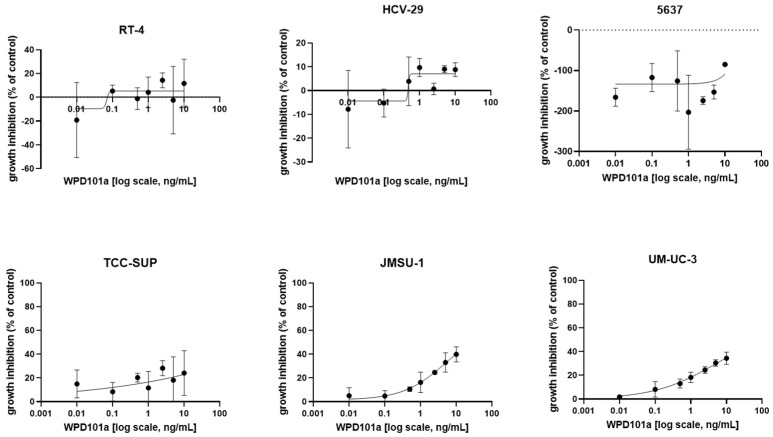
IC_50_ values of WPD101a for BC spheroids. Dose–response curves presented as percentage of inhibition against logarithm of WPD101a concentration in BC spheroids.

IC_50_ values could not be determined for RT-4, HCV-29, and 5637 spheroids, as WPD101a did not exhibit a measurable inhibitory effect on their proliferative activity (Figure 13, Table 3). In TCC-SUP spheroids, a slight dose-dependent trend was observed; however, the variability of the data was too high to allow reliable calculation of the IC_50_ value. In contrast, JMSU-1 and UM-UC-3 spheroids showed clear sensitivity to WPD101a. The IC_50_ value was determined to be 4.860 ng/mL for JMSU-1 and 4.892 ng/mL for UM-UC-3 spheroids, indicating a comparable level of responsiveness to the treatment in these cell lines.

### 2.8. Analysis of the Sensitivity of BC Spheroids to the Immunotoxin WPD101a Performed by Flow Cytometry Using Annexin V and Propidium Iodide (PI) Staining for Cells Grown in 3D Cultures

**Figure 14:**
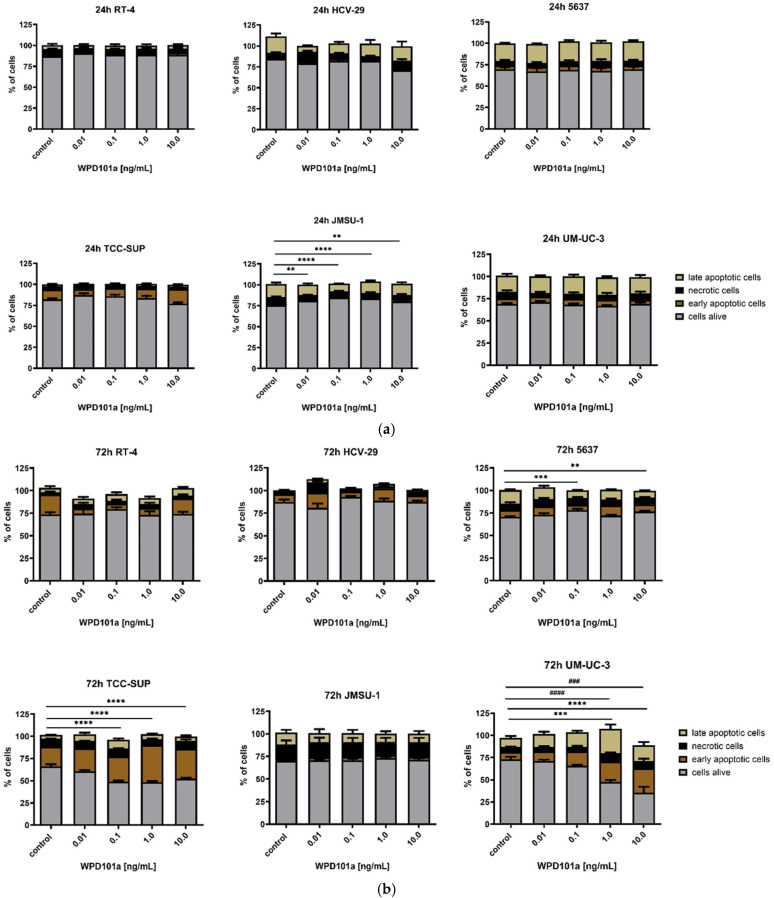
Percentage of early and late apoptotic, necrotic, and viable cells in 3D cultures of BC cell lines treated with the immunotoxin at concentrations of 0.01, 0.1, 1.0, and 10.0 ng/mL, and untreated cells (control) after (**a**) 24 and (**b**) 72 h of exposure to WPD101a. Results are presented as the mean of at least three independent experiments ± SD. Statistical significance for alive (*) and apoptotic (#) cells was determined using one-way analysis of variance (ANOVA) followed by Dunnett’s post hoc test. (*p* < 0.01 (**), *p* < 0.001 (***, ###), *p* < 0.0001 (****, ####).

Analysis of apoptotic cell death revealed that, after 24 h of incubation in 3D cultures, no decrease in the proportion of viable cells was observed in any of the tested BC spheroids at any of the applied immunotoxin concentrations (Figure 14a). Surprisingly, a proportion of viable cells increased in JMSU-1 cells in all tested concentrations. However, prolonged incubation with the immunotoxin for up to 72 h significantly reduced the percentage of viable TCC-SUP cells. The strongest effect was observed at WPD101a concentrations of 0.1 and 1.0 ng/mL, resulting in viability levels of 49% and 48%, respectively, compared to 66% in the control group) (Figure 14b). Furthermore, treatment with 1.0 ng/mL WPD101a led to a statistically significant increase in the proportion of cells in early apoptosis phase, rising to 42% in the treated samples compared to 22% in the control group.

In UM-UC-3 cells, a statistically significant decrease in the percentage of viable cells was observed following treatment with WPD101a at concentrations of 0.10, 1.00, and 10.00 ng/mL compared to untreated controls (66%, 47%, and 36% vs. 73%, respectively). Additionally, treatment with 1.00 ng/mL WPD101a resulted in a significant increase in the proportion of cells in late apoptosis, rising from 10% in the control to 28% in the treated samples. In contrast in the 3D model, JMSU1 cells did not exhibit any response to WPD101a treatment (Figure 14).

#### 2.8.1. WPD 101a Induces Apoptosis-Related Gene Expression in 3D BC Spheroids

**Figure 15:**
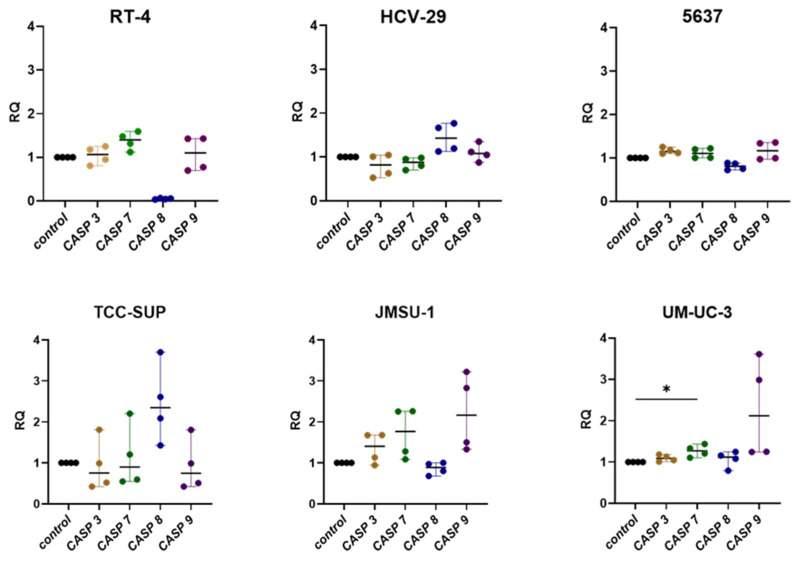
Relative expression of apoptosis-related genes in 3D BC spheroids following WPD101a treatment. Spheroids derived from RT-4, HCV-29, 5637, TCC-SUP, JMSU-1, and UM-UC-3 cell lines were treated with WPD101a (5.0 ng/mL) for 72 h. The relative expression (RQ) of *CASP3*, *CASP7*, *CASP8*, and *CASP9* was determined by qRT-PCR. Gene expression levels were normalized to GAPDH and calculated using the ΔΔCt method, with untreated control spheroids set as 1. Data represents mean ± SD, N ≥ 4; *p* < 0.05 (*).

To assess the expression of apoptosis-related genes, BC spheroids treated with WPD101a at dose of 5.0 ng/mL, selected based on IC_50_ results, were collected after 72 h of incubation and analyzed for expression of *CASP3*, *CASP7*, *CASP8*, and *CASP9* genes (Figure 15). In RT-4 spheroids, substantial variability was observed in the relative expression of caspase genes; however, the mean RQ values ranged from 0.05 for *CASP8* to 1.38 for *CASP7*. Similarly, in HCV-29 spheroids, the relative expression levels of apoptosis-related genes remained close to RQ 1, with the highest increase observed for *CASP8*, reaching a mean value of 1.45. In 5637 spheroids, WPD101a treatment did not induced increased caspase expression, indicating no significant pro-apoptotic effect of the immunotoxin in this cell line. In TCC-SUP spheroids, a marked increase in *CASP8* relative expression was observed, with a mean RQ value of 2.45, whereas the expression levels of the remaining caspases remained close to 1. Analysis of apoptosis-related gene expression revealed that WPD101a exerted the strongest effect in JMSU-1 spheroids, in which the highest upregulation of caspases was observed following treatment. The most pronounced increase was detected for *CASP9* (RQ 2.22), while more moderate elevations were observed for *CASP7* (RQ 1.72) and *CASP3* (RQ 1.36). In UM-UC 3 spheroids treated with WPD101a, increased expression was observed for *CASP9* (RQ 2.27), along with a slight upregulation of *CASP7* (RQ 1.27), whereas no upregulation was detected for the remaining caspases.

## 3. Discussion

Bladder cancer remains a significant unmet medical need, particularly in muscle-invasive bladder cancer (MIBC), where treatment options are limited and associated with considerable morbidity. Despite advances in surgical and systemic therapies, durable and safe targeted treatment strategies are still lacking, especially in advanced disease stages. Therefore, the development of novel, receptor-directed therapeutic approaches is of high importance.

One example of such emerging targeted therapies in BC is epidermal growth factor receptor (EGFR)-targeting immunotoxins, which represent one of the most extensively investigated receptor-directed toxin delivery strategies in this disease [20]. These constructs are fusion proteins composed of a receptor-binding domain, typically an anti-EGFR antibody fragment or ligand, linked to a potent cytotoxic payload derived from bacterial toxins, most commonly Pseudomonas exotoxin A (PE) [20] or diphtheria toxin (DT) [21]. Following receptor-mediated internalization, the toxin moiety enzymatically inhibits protein synthesis, leading to apoptosis. In preclinical studies, EGFR-targeting immunotoxins have demonstrated potent and selective cytotoxicity in EGFR-overexpressing urothelial carcinoma models [20,22]. However, despite their strong preclinical efficacy, their translational potential could be limited by heterogeneous EGFR expression and toxicity concerns in normal tissues, highlighting the need for alternative tumor-specific targets [22].

The major causes of failure in MIBC therapy are tumor heterogeneity, drug resistance, and the presence of CSCs, which can escape chemo- and/or radiotherapy. Therefore, there is an urgent need to develop new therapeutic approaches for MIBC treatment. Targeted therapy that interfere with cellular processes crucial for cancer growth is currently on focus in anti-cancer therapies. As a new target for therapeutic intervention in MIBC, we propose IL-13Rα2, which is expressed in certain types of BC cells but not in normal urothelial cells.

In this context, IL-13Rα2 emerges as a promising candidate due to its more restricted expression pattern in normal tissues and its association with aggressive tumor phenotypes, as confirmed in the present study. The aggressive phenotype of the BC cell model used in our study was confirmed by the expression of CD44 and ALDH1A1, the most biologically and clinically significant CSC markers commonly found in BC metastatic and chemotherapy resistant tumors [23].

IL-13Rα2 is increasingly recognized as an important player in cancer invasion and metastasis, and accumulating evidence support the role of IL-13Rα in mediating IL-13 signaling in cancer cells [24]. Our study demonstrated high levels of IL-13Rα2 expression in the JMSU-1 and UM-UC-3 cell lines, as well as a heterogeneic population of TCC-SUP cells with both high and low IL-13Rα2 expression as confirmed by flow cytometry, WB and immunofluorescence staining. It should also be considered that increased IL-13Rα2 expression may reflect induction of IL-13 signaling in response to inflammation, a hallmark of cancer, thereby promoting tumor cell growth, progression, invasiveness, and metastasis [24].

The first immunotherapeutic intervention in tumors overexpressing IL-13Ra2 involved recombinant immunotoxins, which are chimeric fusion proteins (reviewed by Knudson KM et al. [25]). These fusion proteins bind to surface antigens on cancer cells and induce cells death through receptor-mediated internalization, and cleavage of the toxin moiety within the cytosol, resulting in inhibition of protein synthesis and induction of apoptosis [26,27,28].

In cancer research, 3D models in the form of spheroids offer a simplified and relatively easy-to-reproduce model for studying tumor growth and drug responses. The spheroids were generated from the same BC cell lines used in the 2D experiments, enabling a comparison of the effectiveness of the tested compound in both 2D and 3D models. Spheroids are typically compact and exhibit relatively uniform cell density throughout their surface, with slight morphological differences between the core and the periphery, thereby resembling the 3D architecture of solid tumors. In contrast, the 2D model consists of a monolayer in which the tested substance is in direct contact with all cancer cells. Although spheroids lack the full heterogeneity of native tumors, they can still be used efficiently for drug screening and therapeutic testing [29]. This limitation of the lack of heterogeneity of the 3D model in the current study can be alleviated by using organoids created from primary bladder cancer cells. Organoids better mimic human physiology because they consist not only of tumor cells but also surrounding stromal cells, immune cells (T cells, macrophages), and endothelial cells, and may better reflect the complexity of the tumor microenvironment. Moreover, they maintain spatial organization, cell-to-cell interactions, and oxygen gradients necessary for accurate screening of potential anticancer drugs.

In the present study, IL-13Rα2 activity was inhibited by the immunotoxin WPD101a, as confirmed by functional assays performed in both 2D and 3D models. Functional assessment of BC cells exposed to WPD101a revealed high cytotoxicity and reduced proliferation in BC cell lines expressing IL-13Rα2 in both monolayer and spheroid models. The observed effects varied among BC cell lines and were dependent on IL-13Rα2 expression levels, as well as on dose and exposure time of WPD101a.

To further document the sensitivity of BC cells to WPD101a activity, Annexin V and PI staining, as well as cell cycle analysis, were performed. The results showed that treatment with WPD101a immunotoxin induced apoptosis in IL-13Rα2-expressing BC cells (UM-UC-3, JMSU-1, and TCC-SUP). The percentage of apoptotic cells increased significantly with increasing doses of WPD101a and with prolonged incubation times from 24 to 72 h. In contrast, no such effect was observed in IL-13Rα2-negative cells (5637, RT-4), as confirmed by the absence of a significant increase in cells in the early and late stages of apoptosis.

However, in the HCV-29 cell line, which exhibited very weak expression of IL-13Rα2 detectable only by WB analysis, a slight but nonsignificant increase in apoptotic cells was observed at higher doses of WPD101a after 72 h exposure in the 2D model, but not in the 3D model. This observation further confirms that the specificity of WPD101a activity is associated with receptor density, which directly correlates with intracellular toxin delivery.

Cell cycle analyses showed that the immunotoxin WPD101a did not arrest BC cells in any specific phase of the cell cycle. However, the results confirmed that WPD101a induced apoptosis, as evidenced by a statistically significant increase in the proportion of cells in the subG1 phase. This effect was observed in UM-UC-3 cells as early as after 24 h treatment and became more pronounced over time (up to 72 h). A similar effect was also observed in JMSU-1 and TCC-SUP cells after prolonged exposure.

Differences in sensitivity to apoptotic cell death among IL-13Rα2-positive BC cell lines were clearly associated with IL-13Rα2 expression levels. The highest receptor density was detected in UM-UC-3 cells, followed by in JMSU-1 cells, whereas the lowest expression was in TCC-SUP, as confirmed by MFI analysis. Notably, the high heterogeneity of the TCC-SUP cells, consisting of two populations with high and low IL-13Rα2 expression, also affected the percentage of cells in the subG1 phase. In TCC-SUP cells, a significant increase in the subG1 population was observed only after treatment with the highest tested dose of WPD101a (10.0 ng/mL), whereas UM-UC-3 cells responded to the lowest tested dose (0.01 ng/mL).

Our study revealed that the WPD101a immunotoxin can effectively target BC cells expressing IL-13Ra2 on their surface. The targeted immunotoxin WPD101a induced specific and concentration-dependent death of IL-13Ra2-expressing BC cells through the induction of apoptosis [26,27]. The presence of cells in the early and late phases of apoptosis, together with an increase proportion of cells in the subG1 phase, was further supported by the expression profiles of *CASP3*, *CASP7*, *CASP8* and *CASP9* genes, confirming the efficacy of WPD101a in inducing apoptosis in both 2D and 3D BC models.

In UM-UC-3 cells, the expression of the initiator caspase *CASP8* was significantly increased in the 2D BC model, whereas in the 3D model, apoptosis was associated with upregulation of *CASP9* and *CASP7* expression. Overall, both initiator caspases (*CASP8* and *CASP9*) and effector caspases (*CASP3* and *CASP7*) participated in the apoptotic response induced by WPD101a exposure, and this effect was clearly visible in BC cells expressing IL-13Ra2.

Dose-dependent sensitivity to the WPD101a immunotoxin was also confirmed by Il-13Rα2 expression in BC cells, as demonstrated by MFI assessment and illustrated in the heatmap generated for the 2D model. In the 3D model, the Live/Dead assay showed the penetration of WPD101a into spheroids, as conformed by the presence of dead cells within JMSU-1 and UM-UC-3 spheroids. These results indicate that WPD101a is able to penetrate the spatial structures of the tumor and eliminate cancer cells within the spheroid core.

However, the heterogeneity of IL-13Ra2 expression among bladder tumors may affect the clinical applicability of this therapeutic approach. In this case, monotherapy with the immunotoxin WPD101a would be insufficient because the expression of the target receptor IL-13Ra2 is not uniform throughout the tumor. Cancer cells can frequently increase or decrease IL-13Rα2 expression, allowing the tumor to adapt and build resistance to targeted drugs. Moreover, BC cells with different phenotypes show different responses to conventional treatment which often leads to relapse of disease [24,25].

To overcome the heterogeneity of bladder tumor, several therapeutic strategies are being investigated. Combination approaches are likely necessary to develop a potent antitumor response targeting IL-13Rα2. One approach is to increase the expression level of the target receptor through the concomitant use of chemotherapy (e.g., gemcitabine), which may enhance the antitumor effect by increasing the level of IL-13Rα2 in low expressing tumors. To address the heterogeneity of IL-13Rα2 expression in BC, targeted therapy can be combined with agents targeting different tumor-associated antigens (TAAs). Many promising strategies, including immunotherapy and checkpoint inhibitors targeting proteins such as CTLA-4 and PD-1, may reduce immunosuppression in the tumor microenvironment [25].

Based on these findings, the next stage of research will involve preclinical studies using in vivo model. Prior to in vivo application, we will assess the toxicity profile and determine the therapeutic dose of the WPD101a immunotoxin. These studies are necessary to support the future translation of this agent into clinical trials.

## 4. Materials and Methods

### 4.1. Immunotoxin WPD101a Production in a Bacterial Expression System

The WPD101a (IL-13–DT390) immunotoxin, a recombinant fusion protein composed of the truncated diphtheria toxin domain (DT390) fused to interleukin-13 (IL-13), was produced using a bacterial expression system. The coding sequence was cloned into the pWD-MCS expression plasmid under the control of a T7 constitutive promoter and expressed in *Escherichia coli* BL21(DE3) (New England Biolabs, Ipswich, MA, USA). The plasmid was propagated in DH5α strain (New England Biolabs, Ipswich, MA, USA), and its sequence integrity was confirmed by Illumina MiSeq sequencing (Genomed SA, Warsaw, Poland).

Protein expression was carried out in LB Miller Broth supplemented with 100 mM PBS (pH 7.0), 0.4% glucose, 1 mM MgSO_4_, and 0.2 mg/mL ampicillin. For large-scale production, fed-batch cultivation was performed in a 5 L Biostat A MO UniVessel Glass bioreactor (Sartorius Stedim, Kostrzyn, Poland) under controlled conditions (37 °C, pH > 7.0 adjusted with 0.5 M NaOH, dissolved oxygen > 25%), with induction at mid-exponential phase using 1 mM IPTG. The recombinant protein accumulated predominantly in inclusion bodies. Inclusion bodies were isolated following cell lysis using a buffer containing 0.2 mg/mL lysozyme, 1% Triton X-100, and 0.5 M NaCl. The washed inclusion bodies were solubilized in 100 mM Tris-HCl (pH 7.4) containing 7 M guanidinium hydrochloride and 2 mM EDTA, followed by the addition of DTE to a final concentration of 65 mM. Protein refolding was initiated by rapid dilution into a refolding buffer containing 100 mM Tris-HCl (pH 7.4), 2 mM EDTA, 0.6 M arginine, and 0.9 mM oxidized glutathione, and subsequently continued by dialysis into 20 mM Tris-HCl (pH 8.0) with 100 mM urea. The recombinant protein was purified using a two-step chromatographic process on an ÄKTA Pure 25 M1 FPLC system with Unicorn 7.5.0.1460 software (Cytiva, Marlborough, MA, USA). Clarified dialysate was first subjected to anion-exchange chromatography on a HiTrap Q HP (5 mL) column (Cytiva, Marlborough, MA, USA) using a step gradient in a 20 mM Tris-HCl (pH 8.0)–1 M NaCl buffer system. The protein was further purified by size-exclusion chromatography on a Superdex 200 Increase 10/300 GL column (Cytiva, Marlborough, MA, USA) and stored at −80 °C. The recombinant IL-13–DT390 protein consists of 506 amino acids, with a predicted molecular weight of approximately 55.2 kDa and an isoelectric point (pI) of 5.67. The characteristics of IL-13–DT390 and bioactivity, expressed by affinity for the IL-13Rα2 receptor, and selective cytotoxicity towards the U-251 glioma cell line expressing IL-13Rα2, were presented in previous studies [30].

### 4.2. Bladder Cancer Cell Lines and Reagents

2D cell culture model: Human BC cell lines RT-4, JMSU-1 (DSMZ, Braunschweig, Germany) and HCV-29 (IIET PAS, Wroclaw, Poland) were cultured in RPMI 1640 medium (IIET PAS, Wroclaw, Poland) supplemented with 10% Fetal Bovine Serum (FBS, Thermo Fisher Scientific Inc., Waltham, MA, USA); 5637 (ATCC, Manassas, VA, USA) cell line was cultured in Opti-MEM with GlutaMAX medium (Thermo Fisher Scientific Inc., Waltham, MA, USA) with 3% FBS; UM-UC-3 (DSMZ) cell line was cultured in DMEM medium (IIET PAS, Wroclaw, Poland) with 10% FBS and TCC-SUP (DSMZ) cell line was cultured in DMEM High Glucose (4.5 g/L) medium (IIET PAS, Wroclaw, Poland) with 15% FBS. All media were supplemented with 1% Penicillin-Streptomycin solution and L-glutamine (Thermo Fisher Scientific Inc., Waltham, MA, USA), except Opti-MEM with GlutaMAX medium where only Penicillin-Streptomycin solution was added. Cells were routinely passaged using 0.05% trypsin/0.02% EDTA (*w*/*v*) solution (IIET PAS, Wroclaw, Poland).

For 3D culture, cells were seeded at a density of 1 × 10^4^ cells per well of ultra-low attachment 96-well plates Nunclon™ Sphera™ 3D (Thermo Fisher Scientific Inc., Waltham, MA, USA) and centrifuged at 250× *g* for 5 min. After seeding, plates were incubated in complete growth medium appropriate for each cell line, under standard culture conditions (at 37 °C in a humidified incubator with 5% CO_2_) for 72 h to allow spontaneous aggregation and spheroid formation.

### 4.3. Cell Preparation for All Functional Experiments

For 2D cell culture experiments, BC cells were detached using trypsin solution. For 3D cultures, spheroid-grown cells were dissociated into single-cell suspensions using TrypLE™ Express buffer (Thermo Fisher Scientific Inc., Waltham, MA, USA). Following cell detachment and dissociation, all subsequent experimental procedures were performed identically for both 2D and 3D cultures.

### 4.4. Flow Cytometric Analysis of Surface and Intracellular Protein Expression

The expression levels of selected target molecules were analyzed by flow cytometry using fluorochrome-conjugated or unconjugated antibodies (Ab) as well as the appropriate isotypic controls according to our well establish protocol [31]. Following experimental treatments, cells were harvested and processed for either surface or intracellular staining, depending on the subcellular localization of the target proteins. For the analysis of surface-expressed molecules, cells were collected, washed with phosphate-buffered saline (PBS), and incubated with fluorochrome-conjugated primary Ab directed against the respective surface markers (anti-CD44-PE and anti-CD133-PE; BD Pharmingen, BD Biosciences, San Jose, CA, USA) for 30 min at 4 °C in dark. For IL-13Rα2 detection, cells were incubated with an unconjugated primary Ab (R&D Systems, Minneapolis, MN, USA) for 30 min at 4 °C, followed by staining with a fluorochrome-conjugated secondary Ab (Alexa Fluor 647 donkey anti-goat; Invitrogen, Waltham, MA, USA) for 30 min at 4 °C in dark. For the detection of intracellular proteins, cells were fixed and permeabilized using a commercially available fixation/permeabilization kit (eBioscience, Invitrogen, Waltham, MA, USA) according to the manufacturer’s instructions. Permeabilized cells were subsequently incubated with an unconjugated primary Ab specific for the target intracellular protein ALDH1A1 (Invitrogen, Waltham, MA, USA) for 30 min at 4 °C, followed by staining with a fluorochrome-conjugated secondary Ab (Alexa Fluor 488 goat anti-mouse; Invitrogen, Waltham, MA, USA) for 30 min at 4 °C in dark. After Ab staining, cells were washed to remove unbound Ab and resuspended in an appropriate acquisition buffer. Samples were analyzed using a flow cytometer (FACS Fortessa, Becton Dickinson, East Rutherford, NJ, USA). Fluorescence intensity was quantified as the percentage of positive cells and median fluorescence intensity (MFI) using Flowing Software 2 (Flowing Software ver. 2.5.1., Turku, Finland).

### 4.5. Western Blot

All cells were lysed in RIPA Lysis and Extraction Buffer (Thermo Fisher Scientific, Weston, FL, USA) and stored at −80 °C in the presence of the protease inhibitor cocktails (Promega, Madison, WI, USA). In the next step, the total protein amount in lysed samples was calculated using the BCA Protein Assay Kits (Thermo Fisher Scientific, Weston, FL, USA). Each time 50 µg of total protein was applied per line on SDS-PAGE gels. The WB analysis was performed according to the previously described method [31]. Briefly, cell extracts were loaded on SDS-PAGE and after electrophoresis were transferred onto Immobilon PVDF Membrane (Merck, Darmstadt, Germany). After semi-dry transfer, the membrane was blocked with Every Blot Blocking Buffer (BioRad, Warsaw, Poland) for 5 min at RT. In the next step, the o. n. incubation with primary Ab against human CD133 (Invitrogen, Carlsbad, CA, USA), CD44 (Invitrogen, Carlsbad, CA, USA), ALDH1A1 (Invitrogen, Carlsbad, CA, USA), IL-13Rα2 (R&D Systems, Minneapolis, MN, USA) and β-Actin (13E5 rabbit mAb, Cell Signaling Technology, Danvers, MA, USA) at 4 °C was performed. After washing three times with 0.05% (*v*/*v*) Tween-20 (Thermo Fisher Scientific, Weston, FL, USA) solution in PBS, the membrane was incubated with a secondary HRP conjugate Ab (Azure Biosystems, Dublin, CA, USA) against rabbit (for β-Actin and CD44), mouse (for CD133 and ALDH1A1) and goat (for IL-13Rα2) for 1 h at RT. The chemiluminescent reaction was developed using ECL Western blotting Substrate (Thermo Fisher Scientific, Weston, FL, USA) and visualized using Chemiluminescent Western Blot Imager Azure 300 system (Azure Biosystems, Dublin, CA, USA). A positive controls, lysates from the human HEPC-CB.1 cell lysate were used for CD133, as this cell line is CD133-positive, and the human A549 cell line was used as a control for ALDH1A1 expression.

### 4.6. Cytotoxicity Assay

An in vitro cytotoxicity assay in 2D culture was performed on BC cell lines RT-4, HCV-29, 5637, TCC-SUP, JMSU-1, and UM-UC-3. Cells were seeded in a 96-well plate (TPP, Trasadingen, Switzerland) at the density 4 × 10^3^ cells per well in 100 μL of culture medium a day before the experiment. The next day culture medium was replaced with a fresh one with appropriate concentrations, ranged from 0.01 to 10.0 ng/mL, of the WPD101a immunotoxin (WPD Pharmaceuticals, Warsaw, Poland). After 24, 48, and 72 h, 10% MTT reagent (Sigma-Aldrich, St. Louis, MO, USA) was added to the cells. Following 4 h incubation at 37 °C, 5% CO_2_ medium was removed, and cells were solubilized with 100 μL of DMSO (Th. Geyer, Warsaw, Poland) per well, and absorbance was measured at 570 nm using a Wallac Victor2 microplate reader (Perkin Elmer, Waltham, MA, USA). Results are presented as the mean of at least three independent experiments ± standard deviation (SD), calculated as the percentage of cells relative to the control. Statistical analysis was performed using two-way ANOVA or mixed-effect analysis model with Dunnett’s multiple comparison test. Statistically significant values were defined as *p* < 0.05 (*), *p* < 0.01 (**), *p* < 0.001 (****), and *p* < 0.0001 (****).

### 4.7. Spheroid Treatment with WPD101a

After 72 h of spheroid formation, cultures were treated with the immunotoxin WPD101a at final concentrations of 0 (control); 0.1, 1.0, 5.0, and 10.0 ng/mL. To avoid mechanical disruption of the 3D spheroid structure, only 50% of the culture medium was carefully removed and replaced with fresh medium containing the appropriate concentration of the tested compound. Spheroids were exposed to the WPD101a for 24, 48, and 72 h. All treatments were performed in triplicate within each experiment. Three independent biological experiments were conducted.

Cytotoxicity on BC spheroids RT-4, HCV-29, 5637, TCC SUP, JMSU-1, and UM-UC-3 was evaluated using the MTT assay as described above.

### 4.8. Proliferation Assay in 2D Culture

To evaluate the effect of WPD101a on the proliferation of RT-4, HCV-29, 5637, TCC-SUP, JMSU-1 and UM-UC-3 cell lines in 2D culture, an SRB (Th. Geyer, Warsaw, Poland) assay was performed at 24, 48, and 72 h. Cells (4 × 103 per well in 100 µL of culture medium) were seeded into 96-well plates (TPP, Trasadingen, Switzerland) and incubated for 24 h under standard culture conditions.

After initial incubation, microscopic images were taken to document cell morphology. Subsequently, 50% of the culture medium was replaced with medium containing WPD101a to reach the final concentrations of 0.01, 0.1, 1.0, and 10.0 ng/mL. Untreated cells served as controls. All conditions were tested in triplicate.

Cells were incubated with the tested compounds for 24, 48, and 72 h. At each time point, microscopic images were acquired to assess cell condition. Cell proliferation was then evaluated using the SRB assay according to a standard protocol. Briefly, cells were washed with PBS, fixed with 50% TCA (trichloroacetic acid, Th. Geyer, Warsaw, Poland) for 1 h at 4 °C, washed with water, and air-dried at RT. Plates were then stained with 0.4% SRB solution for 30 min in the dark, washed with 1% acetic acid (Alchem, Torun, Poland) and dried. Protein-bound dye was solubilized with 10 mM TRIS (Th. Geyer, Warsaw, Poland), and plates were shaken for 30 min. Absorbance was measured at 570 nm using a Wallac Victor2 microplate reader (Perkin Elmer, Waltham, MA, USA). Results were expressed as the percentage of cell proliferation relative to untreated controls, data represents mean ± SD, n ≥ 3. Statistical analysis was performed using mixed-effect analysis model with Dunnett’s multiple comparison test. Statistically significant values were defined as *p* < 0.05 (*), *p* < 0.01 (**), *p* < 0.001 (****), and *p* < 0.0001 (****).

### 4.9. Proliferation Assay in 3D Culture

To evaluate the effect of WPD101a on the proliferation of JMSU1, HCV29, RT4, 5637, UM-UC-3, and TCC-SUP spheroids, a CellTiter-Glo^®^ 3D assay was performed at 24, 48, and 72 h post-treatment. Cells (10,000 per well in 100 µL of culture medium) were seeded into 96-well non-adherent plates designed for 3D culture (Nunclon™ Sphera™ 3D, ThermoFisher Scientific, Waltham, MA, USA) and allowed to form spheroids for 72 h under standard conditions. After spheroid formation, 50% of the culture medium was replaced with medium containing WPD101a at concentrations of 0.1, 1.0, 5.0, and 10.0 ng/mL. Untreated cells served as controls. At each time point (24, 48, and 72 h), cell viability was analyzed using the CellTiter-Glo^®^ 3D Assay (Promega, Madison, WI, USA) according to the manufacturer’s instructions. Briefly, 100 µL of CellTiter-Glo 3D Reagent was added to each well of non-adherent plate with spheroids. Then, the contents were mixed vigorously on the shaker for 5 min to induce cell lysis. After mixing, the plate was incubated for 25 min at RT. For luminescence measurement, the entire content of each well (200 µL) was transferred to each well of white 96-well plate for luminescence, and the results were expressed as the percentage of viable cells relative to the untreated control. All experiments were performed independently three times, each in triplicate.

### 4.10. Inhibitory Concentration (IC_50_) Determination

The in vitro cytotoxic activity (IC_50_) of the WPD101a immunotoxin in 2D culture was assessed on the HCV-29, RT-4, 5637, TCC-SUP, JMSU-1, and UM-UC-3 BC cell lines. Cells were seeded in a 96-well plate (TPP, Trasadingen, Switzerland) at the density 4 × 10^3^ cells per well in 100 μL of culture medium a day before the experiment. The next day culture medium was replaced with a fresh one with WPD101a immunotoxin. Cells were then cultured for 72 h, the last day 10% MTT reagent (Sigma-Aldrich, St. Louis, MO, USA) was added to the cells. Following 4 h incubation at 37 °C, 5% CO_2_ medium was removed, and cells were solubilized with 100 μL of DMSO per well, and absorbance was measured at 570 nm. Untreated cells served as a control (100%) and results were calculated as the percentage of inhibition. Results are presented as the mean of at least three independent experiments ± standard deviation (SD).

### 4.11. Annexin V/Propidium Iodide Apoptosis Assay

Apoptosis induction in BC cells following treatment with WPD101a was assessed using the Annexin V/PI double-staining assay (Invitrogen, Carlsbad, CA, USA) combined with flow cytometry. Cells were exposed to WPD101a at concentrations of 0.01, 0.1, 1.0, and 10.0 ng/mL for 24 and 72 h, while cells maintained in culture medium served as controls. Prior to the experiments, cell morphology and confluence were visualized by light microscopy (Primovert, Zeiss, Oberkochen, Germany). Following incubation, cells were trypsinized, washed with PBS, and stained with fluorochrome-conjugated Annexin V and PI according to the manufacturer’s instructions (Dead Cell Apoptosis Kit with Annexin V Alexa Fluor 488 and PI, Thermo Fisher Scientific, Waltham, MA, USA). Fluorescence signals were acquired using a flow cytometer (FACS Fortessa, BD Biosciences, San Jose, CA, USA). Based on Annexin V/PI staining patterns, cell populations were classified as viable (Annexin V^−^/PI^−^), early apoptotic (Annexin V^+^/PI^−^), late apoptotic (Annexin V^+^/PI^+^), or necrotic (Annexin V^−^/PI^+^). The percentage of cells in each population was quantified and compared between treatment groups and time points using Flowing Software 2 (version 2.5.1, Turku, Finland). All experiments were performed in three independent biological replicates, each conducted in two technical replicates.

### 4.12. Cell Cycle Analysis

Cell cycle distribution was analyzed by flow cytometry using the FxCycle™ PI/RNase Staining Solution (Invitrogen, Carlsbad, CA, USA). Following experimental treatments, cells were harvested, washed with PBS, and processed according to the manufacturer’s instructions. Briefly, cells were fixed and permeabilized in cold 70% (*v*/*v*) ethanol and stored overnight at −20 °C to allow DNA staining. Subsequently, cells were incubated with the FxCycle DNA dye to enable quantitative assessment of cellular DNA content. After staining, samples were analyzed using a flow cytometer (FACS Fortessa, BD Biosciences, San Jose, CA, USA). Cell cycle phases were determined based on DNA content, and the proportions of cells in the subG1, G0/G1, S, and G2/M phases were quantified using flow cytometry analysis software (Flowing Software ver. 2.5.1., Turku, Finland).

### 4.13. Caspase Gene Expression by qRT-PCR

BC cells were cultured in a 25T culture bottles (TPP, Trasadingen, Switzerland) at the density 2 × 10^6^ cells per bottle in 3 mL of culture medium a day before the experiment. The next day culture medium was replaced, and cells were cultured for 72 h in the presence of the immunotoxin WPD101a at a concentration of 0.1 ng/mL. Cells were then collected and total cellular RNA was isolated using a NucleoSpin RNA kit (MACHEREY-NAGEL, Düren, Germany). First strand cDNA synthesis was performed by reverse transcription of 0.5 μg of total RNA using the RevertAid First Strand cDNA Synthesis Kit for RT-PCR (Thermo Fisher Scientific Inc., Waltham, MA, USA). Gene expression was assayed by real-time PCR using TaqMan™ Fast Advanced Master Mix for qPCR and specific TaqMan probes: caspase 3 (Hs00234387_m1), caspase 7 (Hs00169152_m1), caspase 8 (Hs04405665_m1), and caspase 9 (Hs00962278_m1) (Thermo Fisher Scientific Inc., Waltham, MA, USA). The reaction was performed in the Applied Biosystems ViiA 7 Real-Time PCR System (Thermo Fisher Scientific, Foster City, CA, USA). GADPH (Hs02786624_g1) was used as the reference gene, and the results were analyzed by the ΔΔCt method and expressed as RQ-Relative Quantification index, with untreated cells used as the control. Results are presented as the mean of at least four independent experiments ± standard deviation (SD). Statistical analysis was performed using Welch’s *t* test. Statistically significant values were defined as *p* < 0.05 (*), *p* < 0.01 (**), *p* < 0.001 (****), and *p* < 0.0001 (****).

### 4.14. Immunofluorescence Analysis of IL-13Rα2 Expression After WPD101a Treatment

The expression levels of IL-13Rα2 were analyzed by immunofluorescence staining of cytospin specimens produced using Cellspin I cytocentrifuge (Tharmac GmbH, Waldsolms, Germany). All cell lines of BC (RT-4, HCV-29, 5637, TCC-SUP, JMSU-1, UM-UC-3) were trypsinised and washed with prewarmed PBS, then cells were seeded at density 5 × 10^3^ cells per 100 µL of PBS per slide. After overnight incubation cytospin specimens were fixed and stained in accordance with the protocol described below. Normal Goat IgG Control (AB-108-C, R&D Systems, Minneapolis, MN, USA) was used as the appropriate isotypic control. Moreover, all BC cell lines (RT-4, HCV-29, 5637, TCC-SUP, JMSU-1, UM-UC-3) were seeded on 10 mm diameter coverslips, and placed in the 24-well plates. Cells were seeded at density 15,000 cells per well and cultured for 24 h in basal culture medium dedicated for each cell line, as described in Section 4.2. After 24 h medium was collected and replaced with fresh basal culture medium alone (control) and supplemented with WPD101a at final concentration 0.1 ng/mL or 1.0 ng/mL. Cells were cultured in the new medium for 72 h. After that time medium was collected, cells were washed with prewarmed PBS and fixed with 4% of paraformaldehyde (PFA) for 20 min. Subsequently, cells were permeabilized with 0.1% Triton X100 (Avantor Performance Materials Poland, Gliwice, Poland) for 15 min at RT and to avoided non-specific Ab binding blocking with 1% bovine serum albumin (BSA, Symbios, Gdansk, Poland) solution was performed for 40 min at RT. Briefly, goat anti-human IL-13Rα2 (AF146, R&D Systems, Minneapolis, MN, USA; final concentration 5 µg/mL) was diluted in antibody diluent (Life Technologies Corporation, Eugene, OR, USA) and 75 µL was applied onto each coverslip and incubated for 2 h at RT. Followed by 3 × 5 min wash in PBS, cells were incubated with mix of conjugated donkey anti-goat IgG (Life Technologies Corporation, Eugene, OR, USA) AF647 diluted at a 1:1000 for 1 h at RT. Finally, coverslips and cytospin specimens were sealed with SlowFade Gold antifade reagent with DAPI (Life Technologies Corporation, Eugene, OR, USA). Finally, cytospin specimens were visualized using an Axio Imager 3 (Zeiss, Jena, Germany), whereas cells on coverslips were visualized using an Axio Observer inverted fluorescence microscope with Apotome 3 (Zeiss, Jena, Germany) and analyzed using Zeiss Zen Blue software, version 3.11.

### 4.15. Statistical Analysis

All statistical analyses were performed using GraphPad Prism version 11 (GraphPad Software, San Diego, CA, USA). For each experiment appropriate test was used as described above.

## 5. Conclusions

The present study demonstrates that the IL-13–DT390 immunotoxin WPD101a induces strong, receptor-dependent cytotoxic effects in IL-13Rα2-positive BC cell lines, whereas IL-13Rα2-negative cells remain largely resistant. Notably, the most pronounced effects were observed in UM-UC-3 and JMSU-1 cells, which exhibited high IL-13Rα2 receptor expression and showed marked reductions in viability, increased apoptosis, and significant accumulation in the subG1 phase in both 2D and 3D models. These findings are consistent with the established mechanism of action of bacterial toxin-based immunotoxins, in which receptor density directly correlates with intracellular toxin delivery and therapeutic efficacy [26]. Importantly, the absence of significant toxicity in IL-13Rα2-negative lines further supports the specificity of WPD101a and reinforces the concept that receptor-restricted expression is critical for selective tumor targeting. Collectively, these results position IL-13Rα2 as a functionally relevant and strategic target in BC therapy using immunotoxins and support the further development of WPD101a as a targeted therapeutic approach.

## Figures and Tables

**Table 1:** Percentage of cells [%] positive for a given marker in 2D model of BC cell lines and mean fluorescence intensity (MFI).

BC Cell Line	CD133	CD44	ALDH1A1	IL-13Rα2
RT-4	0	99 (MFI = 650)	79 (MFI = 348)	0
HCV-29	0	100 (MFI = 580)	93 (MFI = 484)	0
5637	0	95 (MFI = 450)	33 (MFI = 304)	0
TCC-SUP	0	100 (MFI = 550)	50 (MFI = 405)	100 (mean MFI = 7435, MFI IL-13Rα2^low^ = 1150 IL-13Rα2^high^ = 12,000)
JMSU-1	0	100 (MFI = 350)	12 (MFI = 400)	98 (MFI = 8852)
UM-UC-3	0	100(MFI = 430)	84 (MFI = 363)	100 (MFI = 16,587)

**Table 2:** IC_50_ values for WPD101a determined for BC cell lines.

BC Cell Line	IC_50_ WPD101a [ng/mL]
RT-4	not determined
HCV-29	not determined
5637	not determined
TCC-SUP	3.311
JMSU-1	0.002655
UM-UC-3	0.09154

**Table 3:** IC_50_ values for WPD101a determined for BC spheroids.

BC Spheroids	IC_50_ WPD101a [ng/mL]
RT-4	not determined
HCV-29	not determined
5637	not determined
TCC-SUP	not determined
JMSU-1	4.860
UM-UC-3	4.892

## Data Availability

All data generated or analyzed during this study are included in this article.

## Supplementary Materials

The following supporting information can be downloaded at: https://www.mdpi.com/article/10.3390/ijms27125566/s1.

## Abbreviations

The following abbreviations are used in this manuscript:

AP-1	Activator protein 1
BCG	Bacillus Calmette-Guérin
BC	Bladder cancer
CSCs	Cancer stem cells
*CASP*	Caspase
DT	Diphtheria toxin
EGFR	Epidermal growth factor receptor
EAU	European Association of Urology
ERK	Extracellular signal-related kinase
FDA	Food and Drug Administration
GBM	Glioblastoma
IL-13Rα2	Interleukin-13 receptor subunit alpha 2
MIBC	Muscle-invasive bladder cancer
NCCN	National Comprehensive Cancer Network
NMIBC	non-muscle-invasive bladder cancer
PD-1	Programmed death 1
PD-L1	Programmed death ligand 1
PE	Pseudomonas exotoxin A
TNM	Tumor-node-metastasis
qRT-PCR	Quantitative reverse transcription polymerase chain reaction
WPD101a	(IL-13–DT390) immunotoxin
