# Supplementary material for: Immunotoxin WPD101a as a Potential Drug Candidate for Targeted Therapy in Muscle Invasive Bladder Cancer Expressing IL-13Rα2—In Vitro Study
Source: Int J Mol Sci. 2026 Jun 19;27(12):5566. doi: 10.3390/ijms27125566 (PMC13300138; doi:10.3390/ijms27125566)
Supplement: Supplementary file 1 [file ijms-27-05566-s001.zip › ijms-4363920-supplementary.pdf]

Figure S1

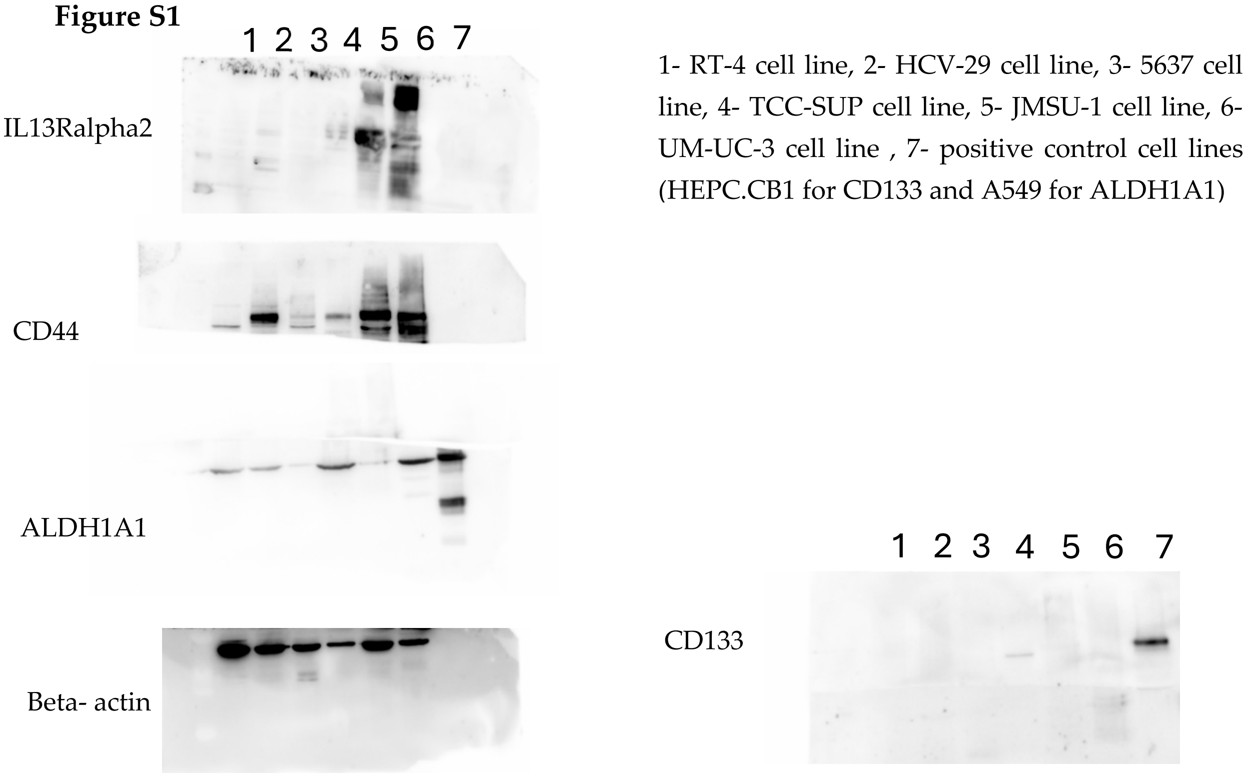

Figure S1. Analysis of the expression levels of IL-13Rα2, CD44, CD133, ALDH1A1, and β-actin in lysates of commercial BC cell lines cultured in a 2D model, determined by WB - chemiluminescent image.

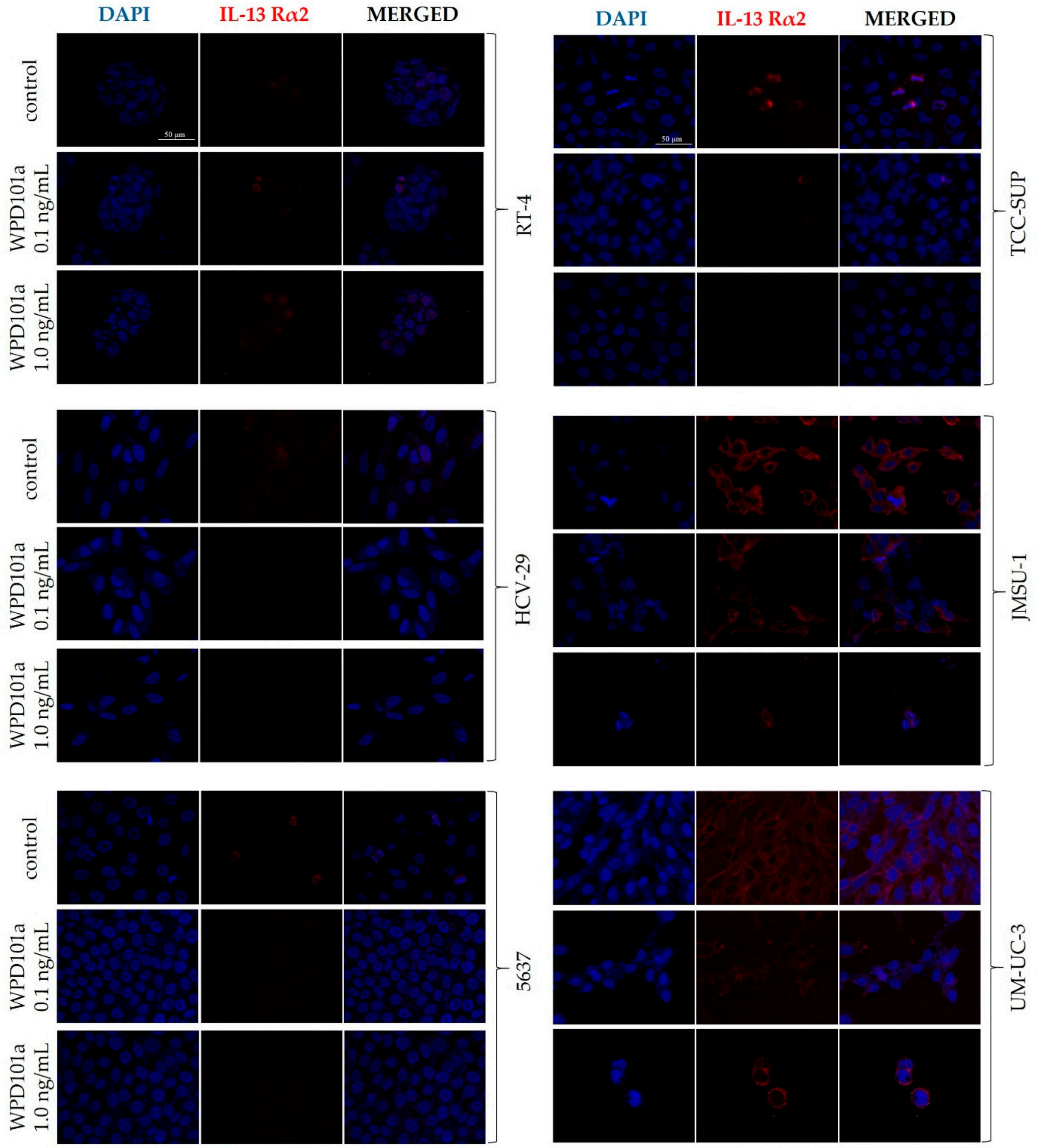

Figure S2. Effect of two WPD101a concentration (0.1 ng/mL and 1.0 ng/mL) on BC cell lines. Immunofluorescence staining showing nuclei (blue, DAPI) and expression of IL-13Rα2 receptor (red, AF647) in RT-4, HCV-29, 5637, TCC-SUP, JMSU-1 and UM-UC-3 cell lines.
